# Mechanistic Characterization of *RASGRP1* Variants Identifies an hnRNP-K-Regulated Transcriptional Enhancer Contributing to SLE Susceptibility

**DOI:** 10.3389/fimmu.2019.01066

**Published:** 2019-05-20

**Authors:** Julio E. Molineros, Bhupinder Singh, Chikashi Terao, Yukinori Okada, Jakub Kaplan, Barbara McDaniel, Shuji Akizuki, Celi Sun, Carol F. Webb, Loren L. Looger, Swapan K. Nath

**Affiliations:** ^1^Arthritis and Clinical Immunology Research Program, Oklahoma Medical Research Foundation, Oklahoma City, OK, United States; ^2^Laboratory for Statistical Analysis, RIKEN Center for Integrative Medical Sciences, Yokohama, Japan; ^3^Department of Rheumatology and Clinical Immunology, Graduate School of Medicine, Kyoto University, Kyoto, Japan; ^4^Department of Statistical Genetics, Osaka University Graduate School of Medicine, Osaka, Japan; ^5^Departments of Medicine, Microbiology and Immunology, University of Oklahoma Health Sciences Center, Oklahoma, OK, United States; ^6^Howard Hughes Medical Institute, Janelia Research Campus, Ashburn, VA, United States

**Keywords:** *RASGRP1*, homology, ERK (extracellular-signal-regulated kinase), genetic variant, luciferase, ChIP-qPCR, EMSA (electrophoretic mobility shift assay)

## Abstract

Systemic lupus erythematosus (SLE) is an autoimmune disease with a strong genetic component. We recently identified a novel SLE susceptibility locus near *RASGRP1*, which governs the ERK/MAPK kinase cascade and B-/T-cell differentiation and development. However, precise causal *RASGRP1* functional variant(s) and their mechanisms of action in SLE pathogenesis remain undefined. Our goal was to fine-map this locus, prioritize genetic variants likely to be functional, experimentally validate their biochemical mechanisms, and determine the contribution of these SNPs to SLE risk. We performed a meta-analysis across six Asian and European cohorts (9,529 cases; 22,462 controls), followed by *in silico* bioinformatic and epigenetic analyses to prioritize potentially functional SNPs. We experimentally validated the functional significance and mechanism of action of three SNPs in cultured T-cells. Meta-analysis identified 18 genome-wide significant (*p* < 5 × 10^−8^) SNPs, mostly concentrated in two haplotype blocks, one intronic and the other intergenic. Epigenetic fine-mapping, allelic, eQTL, and imbalance analyses predicted three transcriptional regulatory regions with four SNPs (rs7170151, rs11631591-rs7173565, and rs9920715) prioritized for functional validation. Luciferase reporter assays indicated significant allele-specific enhancer activity for intronic rs7170151 and rs11631591-rs7173565 in T-lymphoid (Jurkat) cells, but not in HEK293 cells. Following up with EMSA, mass spectrometry, and ChIP-qPCR, we detected allele-dependent interactions between heterogeneous nuclear ribonucleoprotein K (hnRNP-K) and rs11631591. Furthermore, inhibition of hnRNP-K in Jurkat and primary T-cells downregulated *RASGRP1* and ERK/MAPK signaling. Comprehensive association, bioinformatics, and epigenetic analyses yielded putative functional variants of *RASGRP1*, which were experimentally validated. Notably, intronic variant (rs11631591) is located in a cell type-specific enhancer sequence, where its risk allele binds to the hnRNP-K protein and modulates *RASGRP1* expression in Jurkat and primary T-cells. As risk allele dosage of rs11631591 correlates with increased *RASGRP1* expression and ERK activity, we suggest that this SNP may underlie SLE risk at this locus.

## Introduction

Systemic lupus erythematosus (SLE) is a complex autoimmune disease that disproportionately affects people of Asian, African, and Hispanic ethnicities and women, in particular, with higher incidence and disease severity (1). Much of SLE etiology remains mysterious. It has been proposed that complex interactions amongst numerous genes and their products with pathogens and other environmental factors promotes dysregulation of both the innate and adaptive immune responses in SLE. Over 80 SLE susceptibility loci have been identified so far across multiple ethnic groups by genome-wide association studies (GWAS) and candidate gene studies (2, 3). However, the precise underlying variants and functional mechanisms associated with disease are largely unidentified for the vast majority of these SLE-associated signals. Understanding SLE pathogenesis requires identification of true causal variants and the target genes and mechanisms by which they contribute to disease.

Previously, we reported a novel SLE susceptibility signal near the RAS guanyl-releasing protein 1 (*RASGRP1*) in Asians (4). We identified several associated variants, the most significant being an intergenic variant (rs12900339) between *RASGRP1* and *C15orf53* (4). However, the actual predisposing variants, target genes, and underlying mechanisms of action for this region are largely unknown. *RASGRP1* belongs to a family of RAS guanyl nucleotide-releasing proteins (RASGRPs) comprising four members (*RASGRP1* through *RASGRP4*), all with a diacylglycerol (DAG)-binding C1 catalytic domain. Upon antigen stimulation, DAG binding and phospholipase C (PLC) signaling drive RASGRPs to the membrane, where they play important roles in RAS activation (5, 6). *RASGRP1*, originally cloned from the brain (7), was later found highly expressed in T-lymphocytes (8); small amounts of *RASGRP1* expression can also occur in B-lymphocytes, neutrophils, mast cells, and natural killer cells (9–11). *RASGRP1* has been shown to be involved in B-cell development, activation and tolerance, in both mice and humans (12, 13). *RASGRP1*^−/−^ mice have been reported for marked deficiency in thymocyte and lymphocyte development, which was associated with impaired proliferation in response to TCR stimulation (14). Deficiency in *RASGRP1* in mice has been associated with CD4+ and CD8+ T cell lymphopenia (8). However, humans deficient in *RASGRP1* show a decrease in CD4+T concurrent with a relative increase in CD8+T cells (15). *RASGRP1* inhibition impairs T-cell expansion and increases susceptibility to Epstein-Barr virus infection, as well as suppressing proliferation of activated T-cells occurring in autoimmune conditions (16). A recent study reported a heterozygous mutation in *RASGRP1* correlated with autoimmune lymphoproliferative syndrome (ALPS)-like disease (17). *RASGRP1* expression in T-cells also correlated negatively with rheumatoid arthritis disease activity (18). Dysregulated expression of *RASGRP1* has been observed in human SLE. The ratio of normal *RASGRP1* isoforms to isoforms missing exon-11 could be linked to defective poly[ADP-ribose] polymerase 1 (*PARP1*) expression and reduced lymphocyte survival in SLE patients (19, 20). Aberrant splice variants accumulate in SLE patients and adversely affect T-cell function (21). There are conflicting reports of the effect of *RASGRP1* on ERK signaling. On one hand, deficiency in *RASGRP1* expression reportedly decreases ERK phosphorylation in B- and T-cells (15). Hydralazine, a drug that causes drug-induced lupus erythematosus, is reported to inhibit ERK signaling, inducing autoimmunity and the production of anti-dsDNA autoantibodies in mice (22). However, some reports found significantly higher levels of pERK and pJNK in SLE patients with active disease vs. controls and inactive SLE patients (23–25), contradicting earlier reports. In spite of these conflicting reports, the consensus is that *RASGRP1* dysfunction is mechanistically associated with autoimmune phenotypes including SLE.

Here, we fine-mapped an SLE locus near *RASGRP1* that we previously identified (4). Using trans-ethnic meta-analysis across six Asian and European cohorts followed by bioinformatic analyses and experimental validation, we identified potential SLE predisposing variants and defined mechanisms by which these functional variants contribute to SLE pathogenicity.

## Materials and Methods

### Patients and Data

We used all associated SNP data at this locus from six cohorts reported previously (Table 1). We began with our published Asian cohort report [see Supplementary Table 5 in Sun et al. (4)] and augmented this with two publicly available sets of GWAS summary statistics (26, 27) and a partially published Japanese cohort (28). Our original report contained three Asian cohorts (3AS: Korean, Han Chinese, and Malaya Chinese). Japanese samples included samples (456 cases and 1,102 controls) collected under support of the Autoimmune Disease Study Group of Research in Intractable Diseases, Japanese Ministry of Health, Labor and Welfare, and the BioBank Japan Project (28), and added samples obtained at Kyoto University, Japan. SLE classification followed the American College of Rheumatology criteria (29). All sample collections were approved by the Institutional Review Board of the Oklahoma Medical Research Foundation as well as by the collaborating institutions.

**Table 1:** Cohorts used in this study.

**Population**	**Cohort**	**Cases**	**Controls**	**Publication**
Asian	3AS	2,487	7,620	(4)
HC	Han Chinese	1,659	3,398	(26)
EU	European	4,036	6,958	(27)
JAP	Japanese	1,347	4,486	(28) + new Data
TOTAL		9,529	22,462	

*We utilized samples from our previous report (4) (3AS: Korean, Han Chinese and Malaya Chinese) for RASGRP1 SLE association. We added a Han Chinese (HC) and a European (EU) cohort from (26) and a Japanese cohort containing the patients from Okada et al. (28) and additional Japanese samples (JAP)*.

### Quality Control

SNP quality control for our initial Asian cohort has been described elsewhere (4). Quality control for European, Han Chinese 2, and Japanese samples was described in the original publications (26–28). All SNPs in the study were in Hardy-Weinberg equilibrium (*P* > 1 × 10^−6^) and had minor allele frequency >0.5%. Genotypic missingness was <10%. In order to match risk alleles between cohorts, we compared their allele frequencies to the parent populations from the 1,000 Genomes Project. We used the SNP reference dbSNP142 as the SNP-naming convention in common for all variants. SNP imputation for all cohorts was described in their original publications. For this study, SNPs with *r*^2^ and imputation quality information <0.7 were dropped.

### Study Design

**Figure 1:**
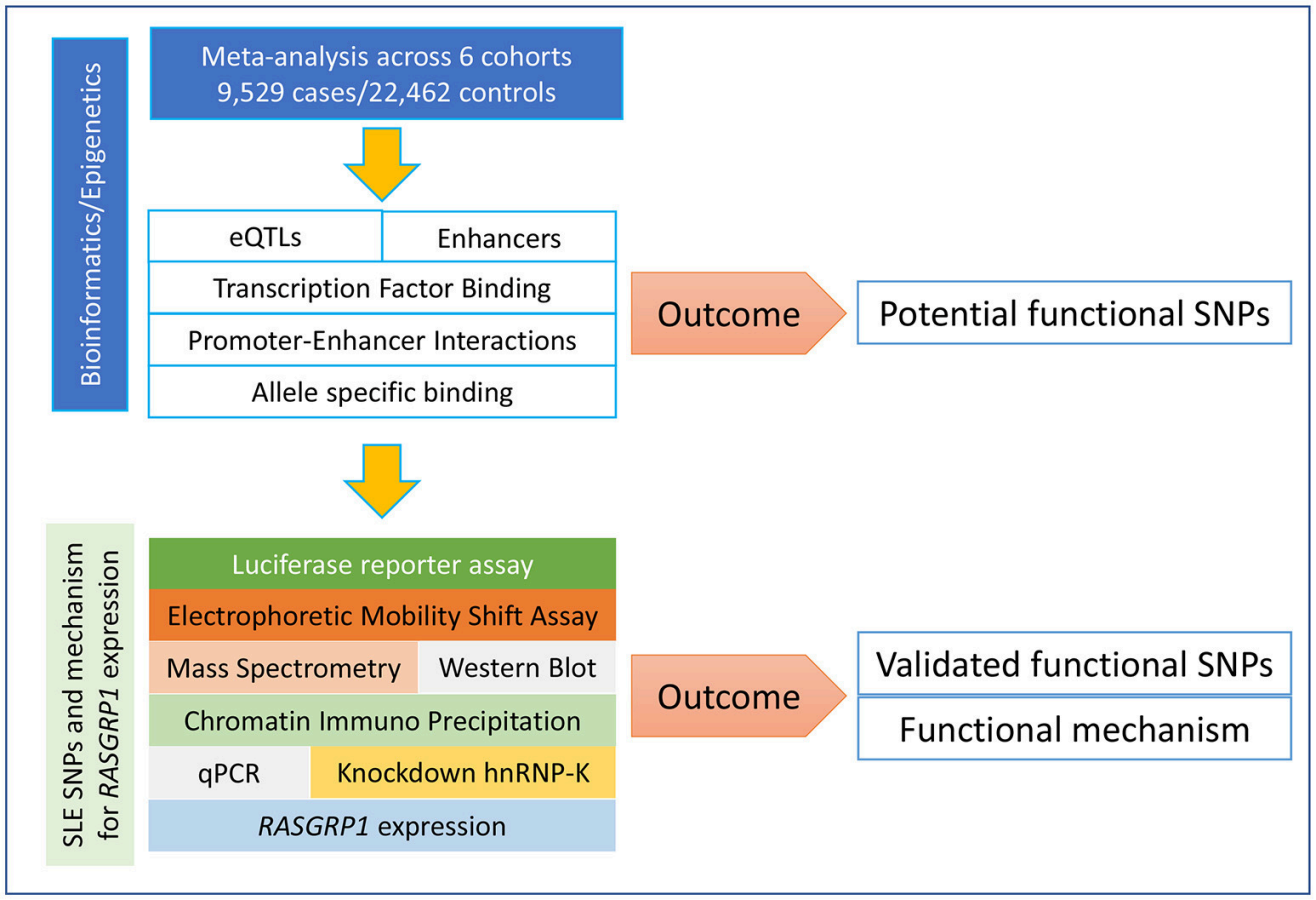
Framework of study design. Our study followed our bioinformatics-prioritized potential functional SNPs with laboratory validation along many different dimensions.

In order to identify *RASGRP1* functional variants and their mechanisms of action, our analysis followed the workflow presented in Figure 1. We first extracted all summary GWAS information in and around *RASGRP1* (118 SNPs) from Supplementary Table 5 in our previous study of Asian SLE (4). We combined these results with a European (27), an Asian (26), and a partially published Japanese cohort (28), to perform meta-analysis. SNPs that passed the genome-wide significant association threshold (*p* = 5 × 10^−8^) were further annotated with functional information. A series of bioinformatics and epigenomic analyses was conducted for each of the candidate SNPs including their effects on gene expression (expression quantitative trait loci, eQTLs), transcription factor binding, promoter/enhancer activities, and chromatin interaction sites. Together, we prioritized and nominated SNPs with stronger association signals and with higher annotated likelihood of being functional (Supplementary Tables 1, 2). Finally, we experimentally validated predicted functions of the nominated SNPs in Jurkat and HEK293 cell lines. Following SNP prioritization, we performed electrophoretic mobility shift assays (EMSAs), followed by mass spectrometry, chromatin immuno-precipitation quantitative PCR (ChIP-qPCR), and inhibition-based expression assays.

### Association Analysis and Trans-Ethnic Meta-Analysis

Association analysis for all cohorts was performed using PLINK (30) and SNPTEST. Meta-analysis for all cohorts was performed in METAL (31) using cohort sample size correction and standard error correction to estimate the 95% confidence interval for odds ratios. Heterogeneity of odds ratios was estimated and informed the use of *P*_meta_ values in the study. Variants with *P*_meta_ < 5 × 10^−3^ were selected for further study.

### Bioinformatic Analysis

Given that candidate SNPs were located in non-coding regions of the genome, we performed a thorough epigenetic annotation of the variants. Initial annotation of epigenetic features was performed in Haploreg (32). Each SNP in the region was collocated with active and regulatory histone marks including H3K27ac, H3K4me1, and H3K4me3, and DNase hypersensitivity sites (DHS) in GM12878, and CD4+ and CD8+ T cells (Supplementary Figure 1). Histone modifications and DHS data were obtained from the ENCODE project (33) and the BLUEPRINT epigenome project (34).

### SNP Prioritization

We used a prioritization algorithm to narrow down the large list of SNPs for further validation. Our strategy consisted of two Bayesian algorithms to score each SNP [3dSNP (35) and RegulomeDB (36)], as well as additional expression, epigenetic, and preferential allele-specific information about each SNP. First, we used the 3dSNP (35) tool to assign functional weights based on the presence of enhancers, promoters, experimentally determined (ChIP-seq) transcription factor binding sites (TFBSs), TFBS motif matching, evolutionary conservation, and presence of 3D chromatin interactions. We assigned a 3dSNP weight of 2 to SNPs >2 standard deviations above the mean, a weight of 1 for scores above the mean, and a weight of 0 for the rest. RegulomeDB (36) scores were also assigned for each candidate SNP and converted to an associated weight. Each functional category, i.e., eQTL, enhancer/super-enhancer, rSNP (37), capture Hi-C, TFBS, and allele-specific expression/binding, was assigned a weight of 1 if the SNP had this feature. Finally, we summed all weights for each SNP and nominated the top SNPs for further experimental validation.

### Expression Quantitative Trait Loci (eQTLs)

All the candidate SNPs were annotated for the presence of eQTLs changing expression of *RASGRP1* and its surrounding genes in multiple tissues. We used expression databases for whole blood (38, 39), immune cell lines (40), and multiple tissues (41) (GTEx Analysis Release V6p). In order to identify quantitative changes in methylation in blood cell lines, we used the WP10 database from the Blueprint epigenome project (42).

### Transcription Factor Binding Sites (TFBSs)

In order to identify allele-specific effects on transcription factor binding (TFBSs), we used the motifBreakr (43) algorithm implemented in R, as well as the PERFECTOS-APE algorithm that identifies fold-changes in binding affinity of SNPs against HOCOMOCO10, HOMER, JASPER, Swiss Regulon, and HT-Selex motif databases. We selected only TFBSs that had at least 5-fold change in affinity.

### Assessing SNP Effects on Enhancer/Promoter Sequences

We assessed whether each SNP was located within regulatory (enhancer/promoter) regions across multiple cell lines using active histone marks (H3K27ac, H3K4me1, and H3K4me3) collocation implemented in the 3dSNP application (35). Super-enhancers were annotated using the dbSuper (44), Prestige (45), and EnhancerAtlas (46) databases.

### Chromatin Interactions

Chromatin looping was identified using capture Hi-C assays obtained from 3D Genome (47), 3DSNP (35) and CHiCP (48); as well as from Promoter-capture Hi-C (49–52) experiments.

### Allele-Specific Binding

Candidate SNPs within the association peaks were further targeted to assess allele-specific binding (ASB) of histone marks H3K4me1 and H3K4me3 in and around them. ASB was calculated using seven heterozygous cell lines (GM10847, GM12890, GM18951, GM19239, GM19240, GM2610, and SNYDER). ASB was implemented in SNPhood (53).

### Luciferase Reporter Assays

To test candidate SNP-containing regions for allele-specific enhancer activity, we cloned all three SNPs (rs1163159, rs7173565-rs7173565, and rs9920715) individually into the enhancer reporter plasmid pGL4.26[luc2/minP/Hygro] (Promega, USA). In brief, genomic DNA from the Coriell cell line having different genotypes for the SNP tested (obtained from NIGMS Human Genetic Cell Repository at the Coriell Institute for Medical Research) was amplified using specific primers containing KpnI and HindIII sites (Supplementary Table 3). These amplified PCR products surrounding rs11631591 (481 bp), rs7170151 (579 bp), and rs9920715 (455 bp) were digested with KpnI and HindIII restriction enzymes and ligated into the pGL4.26 plasmid. After cloning and transformation, the plasmids generated for each genotype were confirmed by direct Sanger DNA sequencing. To study cell type-dependence, we used two different cell types: human embryonic kidney HEK293 and T-lymphoid Jurkat cell lines. HEK293 cells were seeded in 24-well sterile plastic culture plates at a density of 1x10^5^ cells per well with complete growth medium. The cells were transfected with 500 ng of pGL4.26 (with or without insert) along with 50 ng *Renilla* plasmid as control vector to control for differences in transfection efficiency. LipofectAMINE 3000 (Invitrogen, USA) was used for transfection into HEK293 cells, according to the manufacturer's protocol. For Jurkat transfections, we used the Neon Transfection System (Thermo Fisher Scientific). A total of 5 × 10^5^ Jurkat cells was electroporated with a Neon Transfection System (Invitrogen) under the following conditions: voltage (1,050 V), width (30 ms), pulses (Two), 10-μl tip, and Buffer R. For transfection, we used 2 μg of each plasmid containing the insert with risk or non-risk allele, along with 50 ng *Renilla* plasmid. Firefly and *Renilla* luciferase activities were measured consecutively at 24 h after transfection using Dual-luciferase assays (Promega), according to the manufacturer's instructions. Luciferase activity was analyzed with Student's *t*-test implemented in GraphPad Prism7. Differences between relative luciferase activity levels were considered significant if Student's *t*-test *P*-value < 0.05.

### Identification of DNA-Binding Proteins

#### Electrophoretic Mobility Shift Assays (EMSAs) and DNA Pulldown Assays

Jurkat cell lines were obtained from ATCC and maintained in RPMI 1640 medium with 2 mm L-glutamine, 100 μg/ml each of streptomycin and penicillin, and 10% fetal bovine serum at 37°C with 5% CO_2_. Cells were harvested at a density of 8 × 10^5^ cells/ml, and nuclear extracts were prepared using the NER nuclear extraction kit (Invitrogen) with complete protease inhibitors (Roche Diagnostics). Protein concentrations were measured using a BCA reagent. Biotinylated DNA sequence surrounding the candidate SNPs (rs7170151 and rs11631591) was prepared using a synthetic single-stranded DNA sequence (Integrated DNA Technologies, USA) (Supplementary Table 3). Biotinylated DNA sequence with a 5-bp deletion at the SNP region served as a control for the assay. Twenty-five pmol of each DNA product was bound to 1 mg Dynabeads® M-280 Streptavidin (Invitrogen, USA), as per the manufacturer's recommendations. Dynabeads M-280 Streptavidin (Dynal, Inc., Lake Success, NY, USA) were prepared by washing three times in phosphate-buffered saline (pH 7.4) containing 0.1% bovine serum albumin and two times with Tris-EDTA containing 1 M NaCl. Between each wash, beads were pulled down with a Dynal magnetic particle concentrator. Double-stranded, biotinylated oligonucleotides were added to the washed beads, and the mix was rotated for 20–30 min at 21 °C. Equal cpm of proteins translated *in vitro* were diluted to 1× with binding buffer and mixed with ~100 μg of Dynabeads containing 10 pmol of the individual oligonucleotide probe in a final volume of 250 μl. The mixture was rotated at room temperature for 20 min. Proteins bound to the beads were separated from unbound proteins by successive washes, three times with 0.5× binding buffer and once with 1× binding buffer. Higher stringency washes included two washes with 2× binding buffer. Beads and bound proteins were pulled down with a magnetic concentrator, suspended in 1× sample buffer, boiled for 5 min, and resolved on SDS-PAGE gels, followed by peptide mass fingerprint MALDI-MS analysis of single bands.

#### Mass Spectrometry Analysis

Mass spectrometry analysis was performed using a Thermo-Scientific LTQ-XL mass spectrometer coupled to an Eksigent splitless nanoflow HPLC system. Bands of interest were excised from the silver nitrate-stained Bis-Tris gel and de-stained with Farmer's reducer (50 mM sodium thiosulfate, 15 mM potassium ferricyanide). The proteins were reduced with dithiothreitol, alkylated with iodoacetamide, and digested with trypsin. Samples were injected onto a 10 cm × 75 mm inner diameter capillary column packed with Phenomenex Jupiter C18 reverse phase resin. The peptides were eluted into the mass spectrometer at a flow rate of 175 nl/min. The mass spectrometer was operated in a data-dependent mode acquiring one mass spectrum and four CID spectra per cycle. Data were analyzed by searching all acquired spectra against the human RefSeq databases using Mascot (Matrix Science Inc., Boston, MA, USA). Minimum identification criteria required two peptides with ion scores >50% and were verified by manual inspection. We verified the identity of the assayed proteins by Western blot.

### Confirmation of Identified Protein by Western Blot

Mass spectrometry-identified proteins were confirmed by Western blot. Jurkat nuclear extracts after DNA pulldown assay were lysed in sample buffer [62.5 mM Tris·HCl (pH 6.8 at 25°C), 2% wt/vol SDS, 10% glycerol, 50 mM dithiothreitol, 0.01% wt/vol bromophenol blue]. Equal amounts of protein were loaded onto a 10% SDS-PAGE gel (GTX gel BioRad USA). After it resolved, samples were blotted to Nitrocellulose paper using the Trans-blot Turbo Transfer System (BioRad, USA). Membranes were blocked using LI-COR blocking buffer for 2 h and then incubated with primary antibody 1:1,000 dilution (hnRNP-K, Santa Cruz USA) at 4°C overnight, and with a donkey anti-mouse IR-Dye 800 (LI-COR, USA) secondary antibody for 1 h. The membrane was imaged with a LI-COR Odyssey using Auto-Scan. Background-subtracted signal intensity was quantified using Image Studio 4.0 software.

### Chromatin Immuno-Precipitation (ChIP) Assay Followed by qPCR (ChiP-qPCR)

ChIP assays were performed using the MAGnify ChIP system (Life Technologies, NY), according to the manufacturer's protocol. Jurkat cells were fixed for 10 min with 1% formaldehyde to crosslink DNA-protein and protein-protein complexes. The cross-linking reaction was stopped using 1.25 M glycine for 5 min. The cells were lysed, sonicated to shear DNA, and sedimented. Then, their diluted supernatants were incubated with 5 μg hnRNP-K antibody. Ten percent of the diluted supernatants were saved as “input” for normalization. Several washing steps were followed by protein digestion using proteinase K. Reverse crosslinking was carried out at 65°C. DNA was subsequently purified and amplified by quantitative PCR on an SDS 7900 (Applied Biosystems) using specific primers. Because the Jurkat cell line is heterozygous for the SNPs rs11631591 and rs7170151, we performed Sanger DNA sequencing with the ChIP-eluted PCR product.

### Isolation of CD3^+^ T-Cells From Human Blood

We used leukoreduction system chambers (LRS chambers) from human blood donors. LRS chambers were obtained from the Oklahoma Blood Institute (OK, USA) (Supplementary Table 12; Supplementary Figure 9). LRSCs were sterilized externally using 70% (v/v) ethanol and handled in a class 2 laminar flow cabinet. External tubing was cut, the chamber inverted over a 50 ml sterile centrifuge tube (Greiner Bio-One), and the contents allowed to drip through. The contents (usually 20 ml) were then diluted to 90 ml in RPMI medium. The peripheral blood mononuclear cells (PBMCs) were isolated by carefully layering 30 ml fractions over 17 ml of histopaque-1077 (Sigma-Aldrich), which was then centrifuged at 340 g for 45 min at 20°C. The PBMC layer was isolated and washed three times with culture medium with cells centrifuged at 340 g for 15 min for the first wash and 10 min for the subsequent two washes. The isolated PBMCs were counted and viability assessed with Trypan blue using a hemocytometer, then centrifuged at 340 g for 10 min. The untouched CD3^+^ T cells were collected using MojoSort™ Human CD3^+^ T-Cell Isolation Kit, as per manufacturer instructions (BioLegend, San Diego, CA).

### Inhibition of hnRNP-K and ERK Phosphorylation

Inhibition of hnRNP-K was performed in CD3^+^ T cells from healthy controls, as well as in Jurkat T-cells using 5-Fluorouracil (5-FU) (Sigma Aldrich, USA), as described previously (54). Isolated CD3^+^ T-cells and Jurkat cells were cultured in RPMI-1640 medium containing 10% heat-inactivated fetal bovine serum (Invitrogen) and kept at 37°C in 5% CO_2_ conditions. For 5-FU treatment, the drug was first dissolved in dimethyl sulfoxide (DMSO) and further diluted in medium before use. Cells were treated with 20 ng/μl 5-FU, unless otherwise stated. Next, to examine whether hnRNP-K and/or *RASGRP1* down-regulation by 5-FU led to inhibition of EKR phosphorylation of ERK, Jurkat and CD3^+^ T-cells were pretreated with PMA 5 μg/μl for 30 min, prior to drug (5-FU) treatment. Inhibition of hnRNP-K and *RASGRP1* was detected using mRNA expression analysis with quantitative PCR (after 48 h) and by Western blot (after 72 h).

## Results

### Patients and Samples

We used five Asian cohorts and one cohort of European descent; sample sizes for the meta-analysis were 9,529 SLE cases and 22,462 controls (Table 1).

### Fine-Mapping, Replication and Meta-Analysis of *RASGRP1* Association

**Figure 2:**
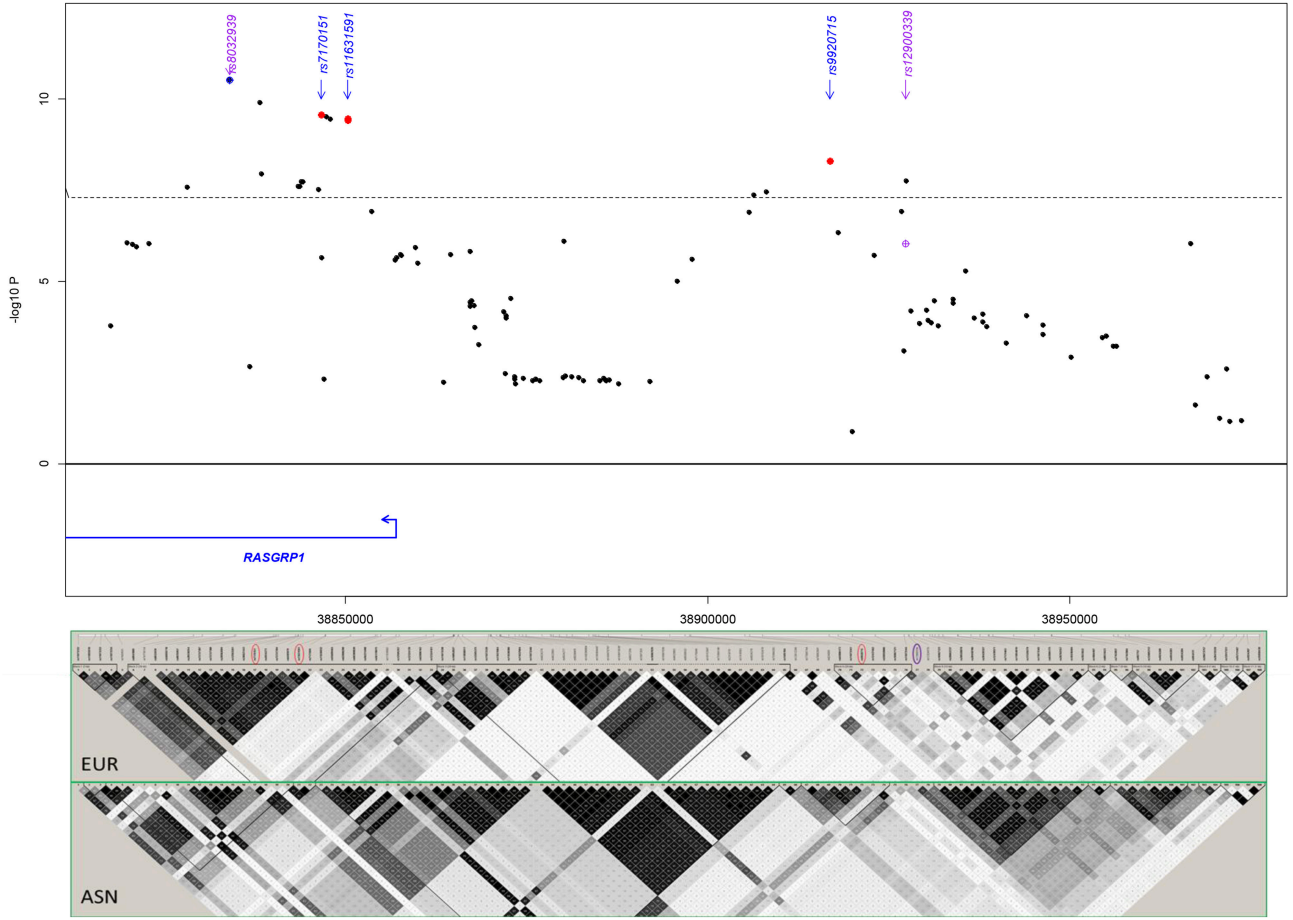
Meta-analysis in the *RASGRP1* region. Blue diamond: lead SNP rs8032939 following initial meta-analysis. Red circles: SNPs chosen for experimental validation. rs11631591-rs7173565 are considered together due to their proximity; only rs11631591 is labeled. Purple diamond: our previously reported (4) lead SNP rs12900339. Linkage disequilibrium in the region (bottom) is notably different between European (EUR) and Asian (ASN) populations.

First, we probed our previously reported SLE-associated region (chr15: 38.4–39.2 MB, hg19) and extracted association results for six cohorts from the region containing the genes *RASGRP1* (RAS guanyl-releasing protein 1, a diacylglycerol-regulated guanine nucleotide exchange factor) and *C15orf53* [encoding a protein of unknown function linked to alcohol dependence (55)]. The strongest association signal among Asian cohorts localized to intron 2 of *RASGRP1* (Figure 2; Table 2). Meta-analysis with all cohorts identified the largest signal at intronic SNP rs8032939 [*P*_meta_ = 3.2 × 10^−11^, OR (95%CI) = 0.88 (0.85–0.92)]. We identified 17 genome-wide significant (GWS) SNPs (*P*_meta_ < 5 × 10^−8^). Our previously reported lead SNP rs12900339 (4) did not reach GWS (*P*_meta_ = 9.2 × 10^−7^) (Table 2). Analysis of the association signals in the context of linkage disequilibrium (LD) of 1,000 Genome populations (EUR, ASN; Figure 2) identified two uncorrelated association signals (Supplementary Table 1). The main signal occurred at rs8032939 in intron 2 (Figure 2), while the second signal localized to the intergenic region between *RASGRP1* and *C15orf53*: SNP rs9920715 [60 kb 5′ of *RASGRP1*; *P*_meta_ = 5.1 × 10^−9^; OR (95%CI) = 0.89(0.86–0.93)]. Many (27 of 118 SNPs) variants were intronic (Figure 2). We then examined the 18 GWS SNPs with bioinformatic and epigenomic analysis (Table 2). Our top SNP (rs8032939) was previously reported as a rheumatoid arthritis (RA)-associated SNP (56). Within the intronic signal, we also identified rs8035957 (*P*_meta_ = 1.3 × 10^−10^), associated with Type I Diabetes (57).

**Table 2:** Meta-analysis of the *RASGRP1* region.

**SNP**	**Nearby gene**	**Function**	**Position**	**A1**	**A2**	^*^3AS (4)	**CH (26)**	**EU (27)**	**JAP (28)**	**Meta-analysis**				
						***P*-value**	***P*-value**	***P*-value**	***P*-value**	***P*-value**	**OR**	**95%CI**	**HetPVal**	**Direction of OR**
rs8043085	RASGRP1	intron 2	38,828,140	T	G	3.91E-03	5.53E-01	1.02E-03	4.52E-05	2.68E-08	1.11	1.1-1.16	0.3072	++++++
rs8032939	RASGRP1	intron 2	38,834,033	T	C	1.40E-04	5.81E-02	1.03E-03	4.35E-05	3.16E-11	0.88	0.85-0.92	0.4913	−−−−−−
rs8035957	RASGRP1	intron 2	38,838,264	T	C	2.52E-04	4.89E-02	1.40E-03	5.14E-05	1.31E-10	0.89	0.85-0.92	0.5035	−−−−−−
rs28536554	RASGRP1	intron 2	38,838,432	T	A	6.37E-03	3.13E-01	9.72E-04	1.72E-05	1.15E-08	0.89	0.9-0.93	0.3438	−−−−−−
rs72727387	RASGRP1	intron 2	38,843,476	A	G	3.43E-03	2.71E-01	3.65E-03	3.92E-05	2.54E-08	1.11	1.07-1.15	0.4756	++++++
rs72727388	RASGRP1	intron 2	38,843,694	C	T	3.36E-03	2.70E-01	3.67E-03	3.92E-05	2.55E-08	0.90	0.9-0.93	0.4756	−−−−−−
rs28582094	RASGRP1	intron 2	38,843,887	G	A	3.77E-03	2.62E-01	2.34E-03	4.16E-05	1.87E-08	0.90	0.87-0.93	0.4971	−−−−−−
rs12593201	RASGRP1	intron 2	38,844,106	A	G	3.25E-03	2.62E-01	2.91E-03	3.92E-05	1.90E-08	1.11	1.1-1.15	0.4893	++++++
rs36027443	RASGRP1	intron 2	38,846,347	A	G	3.79E-03	2.78E-01	2.69E-03	3.63E-05	3.08E-08	1.11	1.07-1.15	0.4629	++++++
rs7170151	RASGRP1	intron 2	38,846,678	C	T	3.36E-04	5.28E-02	2.72E-03	5.93E-05	2.69E-10	1.13	1.1-1.17	0.4429	++++++
rs6495979	RASGRP1	intron 2	38,847,359	C	T	3.21E-04	6.55E-02	3.02E-03	7.01E-05	3.18E-10	1.13	1.09-1.17	0.3495	++++++
rs11348849	RASGRP1	intron 2	38,847,877	I	D	3.30E-04	6.84E-02	3.71E-03	6.20E-05	3.62E-10	1.13	1.07-1.22	0.3029	++++++
rs11631591	RASGRP1	intron 2	38,850,262	T	C	2.53E-04	7.00E-02	3.49E-03	6.51E-05	3.43E-10	0.89	0.9-0.92	0.2393	−−−−−−
rs7173565	RASGRP1	intron 2	38,850,330	T	C	2.83E-04	7.01E-02	3.50E-03	6.51E-05	3.79E-10	0.89	0.9-0.92	0.2677	−−−−−−
rs62006173	RASGRP1-C15orf101	intergenic	38,906,396	T	C	2.06E-04	1.04E-02	1.54E-01	1.94E-03	4.50E-08	0.87	0.8-0.92	0.6036	−−−−−−
rs11073341	RASGRP1-C15orf102	intergenic	38,908,135	G	A	1.38E-04	9.76E-04	1.28E-01	8.25E-04	3.67E-08	1.11	1.1-1.16	0.04208	++++++
rs9920715	RASGRP1-C15orf103	intergenic	38,916,906	T	C	4.21E-05	1.10E-03	5.90E-02	1.34E-03	5.11E-09	0.89	0.86-0.93	0.06495	−−−−−−
rs12900339	RASGRP1-C15orf109	intergenic	38,927,386	G	A	2.75E-06	3.39E-01	3.28E-01	2.69E-04	9.19E-07	1.09	1.1-1.14	0.0166	++++++
rs12324579	RASGRP1-C15orf110	intergenic	38,927,510	C	G	1.20E-05	8.56E-02	3.00E-01	1.09E-05	1.80E-08	0.90	0.9-0.93	0.02404	−−−−−−

*We identified 18 genome-wide significant SNPs in RASGRP1 (intron 2) and between RASGRP1 and C15orf53 (intergenic)*.

* *Sun et al. (4) Supplementary Table 5 (Korean, Han-Chinese, Malay Chinese, 3AS) was used as the discovery cohort and was replicated in Morris et al. (26) (Han Chinese, HC); and Bentham et al. (27) (European, EU) and in Okada et al. (28) + new samples (Japanese, JAP) cohorts. OR, Odds ratio; 95% CI, 95% confidence interval; HetPVal, P-value for heterogeneity meta-analysis test. Direction of OR is presented as + if OR > 1 and—if OR <1. Note that all OR directions are consistent for all SNPs*.

### Evaluating Functional SNPs

To identify putative functional SLE SNPs in and around *RASGRP1*, we computed weighted scores for each SNP by integrating multiple sources of functional annotation, including allele-dependent gene expression, overlap with annotated enhancers and promoters, binding affinity to transcription factors, and collocation with anchors in promoter-enhancer-capture Hi-C experiments (Supplementary Table 2).

### Gene Expression

We then identified allele-dependent changes in gene expression by annotating SNPs using expression quantitative trait locus (eQTL) databases for multiple tissues (**Methods**). All candidate LD SNPs were eQTLs in blood cell lines (3.2 × 10^−3^ > *P* > 1.9 × 10^−4^; Supplementary Table 4), as well as in skin, esophagus, and testis (Table 3). The intronic (main signal) SNPs affected expression of both *RASGRP1* and *C15orf53*, while the intergenic (secondary) SNPs (in LD with rs9920715) altered expression of only *RASGRP1*. *RASGRP1* SNPs also affected expression of long non-coding RNAs (lncRNAs) *RP11-102L12.2* and *RP11-275I4.2* in non-blood cell lines. All eQTL risk alleles increased expression of *RASGRP1* in multiple cell lines (Supplementary Table 4; Supplementary Figure 2), but had opposing effects on the neighboring gene *C15orf53* (Supplementary Table 4). We also found significant effects of two linked SNPs (rs11073344, rs11631591) on methylation of *RASGRP1* in T-cells and neutrophils, respectively (Supplementary Table 5).

**Table 3:** Relevant epigenetic features of genome-wide significant SNPs.

**SNP**	**Nearby gene**	**Function**	**Position**	**3D_score**	**RegulomeDB score**	**Weight 3d score**	**Weight regulome**	**eqtl**	**Enhancer**	**rSNP**	**PCHiC**	**TFBS**	**ASE/ASB**	**Total**
rs8043085	RASGRP1	intron 2	38,828,140	25.72	5	2	2	1	1	1	1	1	0	9
rs8032939	RASGRP1	intron2	38,834,033	4.67	7	0	0	3	1	1	1	1	1	8
rs8035957	RASGRP1	intron2	38,838,264	1.85	7	0	0	4	1	1	0	1	1	8
rs28536554	RASGRP1	intron2	38,838,432	2.12	6	0	1	2	1	1	0	1	1	7
rs72727387	RASGRP1	intron2	38,843,476	3.55	6	0	1	2	2	1	0	1	1	8
rs72727388	RASGRP1	intron2	38,843,694	3.56	7	0	0	2	2	1	0	1	1	7
rs28582094	RASGRP1	intron2	38,843,887	4.04	6	0	1	2	2	1	0	1	1	8
rs12593201	RASGRP1	intron2	38,844,106	5.49	7	0	0	3	2	1	1	1	1	9
rs36027443	RASGRP1	intron2	38,846,347	11.23	5	1	2	2	2	0	1	1	0	9
**rs7170151**	RASGRP1	intron2	38,846,678	20.04	3a	2	4	3	2	1	1	1	1	**15**
**rs6495979**	RASGRP1	intron2	38,847,359	8.79	7	1	0	3	2	1	1	1	1	**10**
rs11348849	RASGRP1	intron2	38,847,877	8.09	7	1	0	0	2	1	1	0	0	5
**rs11631591**	RASGRP1	intron2	38,850,262	6.86	3a	1	4	2	2	1	1	1	1	**13**
**rs7173565**	RASGRP1	intron2	38,850,330	8.41	4	1	3	4	2	1	1	1	1	**14**
rs62006173	RASGRP1-C15orf53	intergenic	38,906,396	2.36	6	0	1	0	0	0	0	1	3	5
rs11073341	RASGRP1-C15orf53	intergenic	38,908,135	1.26	5	0	2	3	0	0	0	1	3	9
**rs9920715**	RASGRP1-C15orf53	intergenic	38,916,906	7.83	3a	1	4	3	0	0	1	1	2	**12**
rs12900339	RASGRP1-C15orf53	intergenic	38,927,386	0.81	6	0	1	3	0	0	0	1	3	8
rs12324579	RASGRP1-C15orf110	intergenic	38,927,510	1	7	0	0	1	0	0	0	0	0	1

*We integrated scores from 3dSNP, RegulomeDB and rSNP with blood cell-specific information for eQTLs, enhancer/super-enhancer existence, promoter capture HI-C (PCHiC), transcription factor binding site (TFBS) disruption and allele-specific expression/binding (ASE/ASB) into a weighted score for SNP prioritization. We chose the top three SNPs for further validation (rs11631591 and rs7173565 were used together because of the short distance between them). The top five SNPs are presented in bold*.

### Overlap With Enhancers and Super-Enhancers

Then, we investigated the potential of the candidate SNPs to act as enhancers of *RASGRP1* expression. Three GWS SNPs (rs6495979, rs11631591, and rs7173565) overlapped with ENCODE-annotated enhancers for *RASGRP1* in lymphoblastoid cells (GM12878, GM12892) and also in CD8^+^ T-cells. These three GWS SNPs (all intronic) localized to super-enhancers [i.e., collections of multiple contiguous enhancers (58)] for *RASGRP1* in CD4^+^ CD25^−^ CD45RA^+^ naïve cells, CD4^+^ CD25^−^ CD45RO^+^ memory cells, CD8^+^ primary cells, CD4^+^ CD25^−^ Il17^+^ phorbol myristate acetate (PMA)-stimulated Th17 cells, and CD4^+^ CD25^−^ Il17^−^ PMA-stimulated Th17 cells (Supplementary Table 6). This suggests that these SNPs may regulate *RASGRP1* in T-lymphocytes.

### Chromatin Interactions

Since all candidate SNPs reside outside of the *RASGRP1* promoter, we investigated if the SNPs overlapped with anchors in promoter-enhancer connections through chromatin interactions. We used promoter-capture Hi-C data on blood cell lines, in particular T-cells, to identify physical interactions between the intronic signal and the *RASGRP1* promoter (Supplementary Table 7; Supplementary Figure 3). We also examined the physical interaction between the intergenic region (represented by rs9920715) and the promoters of *RASGRP1* and *C15orf53*. We identified multiple significant promoter-enhancer interactions between the intronic signal and *RASGRP1, C15orf53, FAM98B*, and *SPRED1*, and multiple interactions between the intergenic signal and the promoter of *RASGRP1* (Supplementary Table 7).

### Effect on Cytokine Production

A critical feature in SLE pathogenicity is cytokine production (59); thus, we investigated if these SNPs alter cytokine abundance. Our candidate SNPs significantly increased expression of interleukins IL6 and IL22 and tumor necrosis factor (TNFα), while SNP rs9920715 exclusively increased IL22 expression (Supplementary Table 8).

### Allele-Specific Binding

We found that 14 of the candidate GWS SNPs also had allele-specific binding (ASB) to H2K27ac in monocytes, neutrophils, and T-cells (Supplementary Table 9), while rs9920715 showed ASB with H3K4me1 in T-cells and neutrophils. To characterize the regulatory mechanisms involved, we assessed ASB of histone marks H3K4me1 and H3K4me3 at and around candidate SNPs (Supplementary Table 9; Table 3). We identified a significant regulatory region associated with promoter mark H3K4me3 with a higher binding affinity to the extended region (~1 kb) containing the risk alleles (both C) of intronic SNPs rs11631591-rs7173565 (Supplementary Figure 4a). In addition, we identified marginally significant ASB to enhancer mark H3K4me1 at SNPs rs6495979 and rs7170151, which tagged a regulatory region within ~500 bp (Supplementary Figure 4b). These data indicate that allele-specific differences might affect chromatin interactions.

### Validation of Enhancer by Luciferase Assays

**Figure 3:**
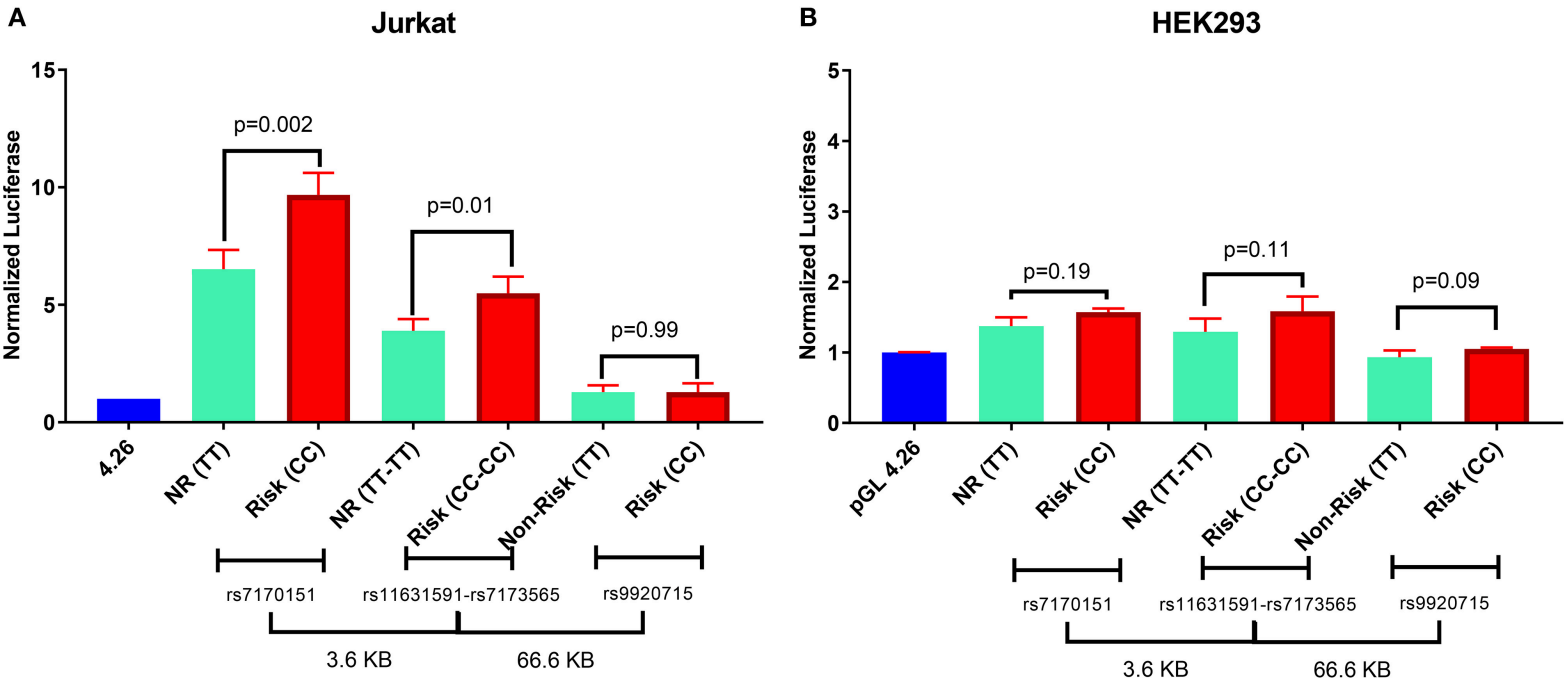
Luciferase reporter assay for rs7170151, rs11631591-rs7173565 and rs9920715. **(A)** Jurkat cells. **(B)** HEK293 cells. Empty vector pGL4.26 was used as reference. NR: non-risk. *P*-values are for Student's *t*-test.

When testing in a luciferase reporter assay, rs7170151 and rs11631591 showed marked (up to 10-fold over empty vector) enhancer activity in Jurkat cells (*P* = 3.0 × 10^−4^, *P* = 1.0 × 10^−3^, respectively) and less so (1.6-fold) in HEK293 cells (*P* = 4.0 × 10^−2^, *P* = 3.0 × 10^−3^); on the other hand rs9920715 functioned as a very weak enhancer only in HEK293 (*P* = 4.1 × 10^−2^) (Figure 3). Furthermore, rs7170151 and rs11631591 showed dramatic allelic differences in enhancer function. Genomic regions containing homozygous risk alleles of rs7170151 (C) and rs11631591 (C) showed significantly higher enhancer activity (~50% increase; *P* = 1.0 × 10^−2^ and *P* = 2.3 × 10^−3^, respectively; Figure 3A) compared to non-risk alleles, but only in Jurkat cells. This allele-dependent enhancer activity is consistent with the allele-specific expression we observed in the eQTL data. There were no significant differences in HEK293 cell lines (Figure 3B), suggesting that enhancer activity depends on white blood cell-specific factors. The third intergenic SNP (rs9920715) did not show enhancer activity in any assayed cell type (Figures 3A,B).

### Transcription Factor Binding

We next assessed allele-specific changes in transcription factor binding site (TFBS) affinity using five motif databases (**Methods**). We identified 256 TFBSs significantly affected by ten of our SNPs (Supplementary Table 10). Notably, we found 43-fold higher affinity of promoter-specific TF YY1 to the non-risk allele (T) of rs7173565 and 42-fold higher affinity of TF GATA (GATA1.3.p2 motif) to the risk (T) allele of rs6495979. Interestingly, SLE-risk ETS1 (60) binding had 10-fold higher affinity to the risk (C) allele of rs7173565, while SLE-risk IRF5 (61) bound 6-fold more tightly to the non-risk (C) allele of rs6495979.

### Identification of DNA-Binding Proteins

We detected DNA-binding protein complexes using electrophoretic mobility shift assays (EMSAs) and DNA pulldown assays using a 41 bp-long dsDNA containing the rs11631591-rs7173565 (homozygous risk, CC; or homozygous non-risk, TT) alleles (Supplementary Table 11). We prepared nuclear extracts from Jurkat cells and incubated them with biotin-labeled dsDNA (risk vs. non-risk) bound to magnetic beads coated in streptavidin. EMSA showed multiple bands of DNA-bound proteins (Supplementary Figure 5). We observed allele-specific binding of a protein complex at 75 kDa. Although EMSA is not a quantitative assay, we observed in multiple independent experiments that the intensity of the band with the risk (CC) oligo was darker than with the non-risk (TT) oligo, suggesting allele-specific differential binding (Supplementary Figure 5). Using mass spectrometry analysis of bound proteins, we identified heterogeneous nuclear ribonucleoprotein K (hnRNP-K) isoform b as the most abundant bound protein (Supplementary Table 11). hnRNP-K was also the protein whose binding was most diminished by substitution of the risk CC by non-risk TT nucleotides. We also confirmed that the identified protein bound with the risk oligo for the region of rs11631591 was hnRNP-K through EMSA followed by Western blot (Supplementary Figure 6).

### SNPs Bind to Different Transcription Factors in an Allele-Specific Manner

**Figure 4:**
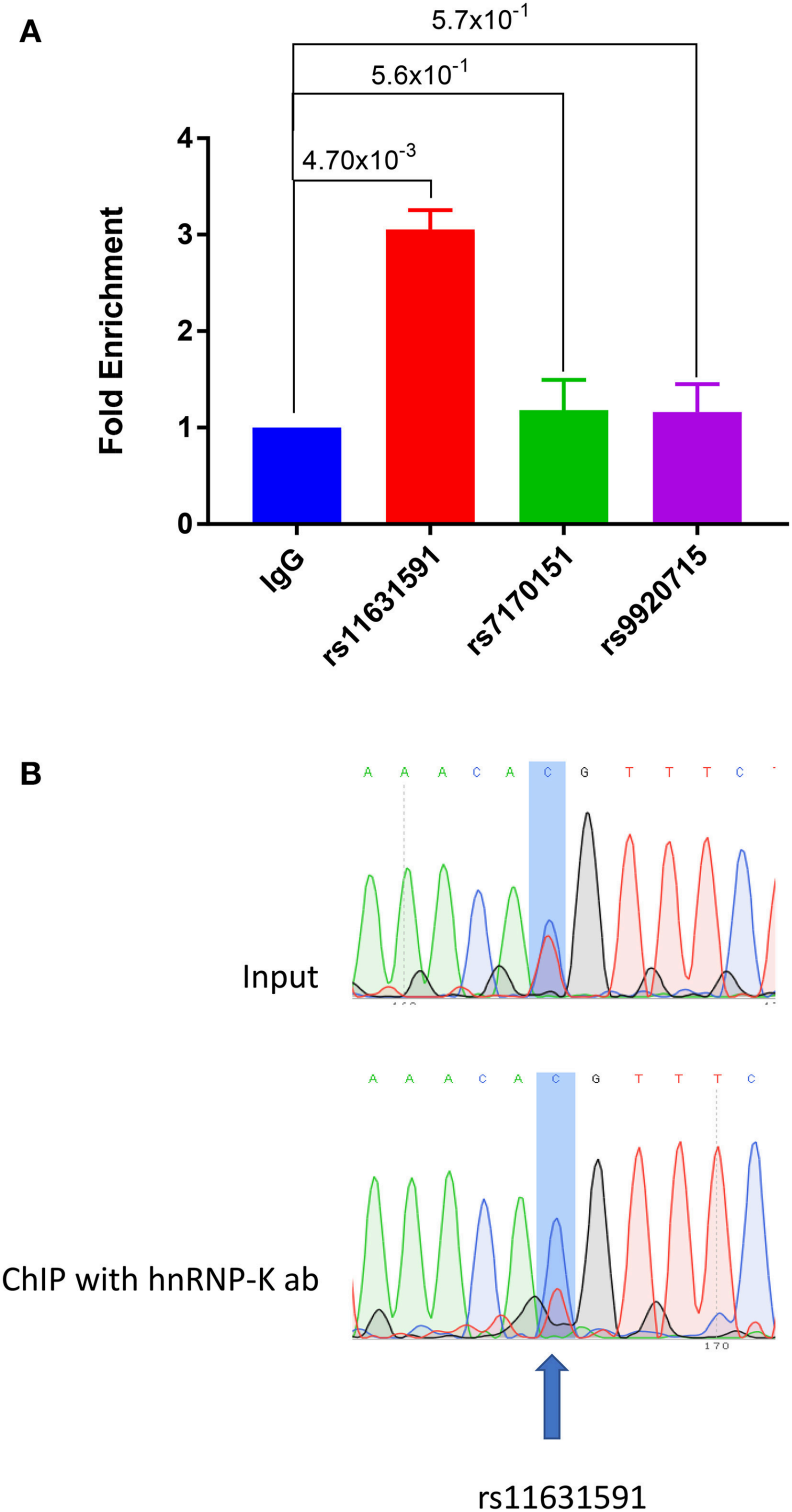
**(A)** ChIP-qPCR of sequences containing SNPs rs11631591-rs7173565, rs7170151 or rs9920715 in Jurkat cells. SNP rs11631591 showed 3-fold enrichment of hnRNP-K binding over IgG control. No significant enrichment at the other two SNPs was observed. *P*-values are for Student's *t*-test. **(B)** Sequence chromatographs from a heterozygous sample at rs11631591 showing difference in binding between the input (equal binding to the two alleles, above) and the ChIP assay at the risk allele (2–3× more binding to the risk C allele, below).

Using EMSA and mass spectrometry, we showed that hnRNP-K protein has tighter binding affinity to the risk genotype (CC) of SNP rs11631591-rs7173565. We validated these findings using Jurkat (heterozygous CT at rs11631591-rs7173565) to perform chromatin-immunoprecipitation (ChIP) followed by RT-qPCR (ChIP-qPCR). We observed significant enrichment in binding of the hnRNP-K antibody to the SNP region of rs11631591, but did not observe any binding of hnRNP-K antibody to either rs7170151 or rs9920715 (Figure 4A). To determine preferential or allele-specific binding, we performed Sanger sequencing on the region containing rs11631591-rs7173565. Both alleles were present in the original input sample; however, only the risk allele (C) was detected significantly higher than the non-risk allele (T) in chromatograms of the ChIP-eluted PCR product (Figure 4B). These data suggest preferential allele-specific binding of the rs11631591-rs7173565 risk locus to hnRNP-K.

### hnRNP-K Plays an Important Role in *RASGRP1* Expression

**Figure 5:**
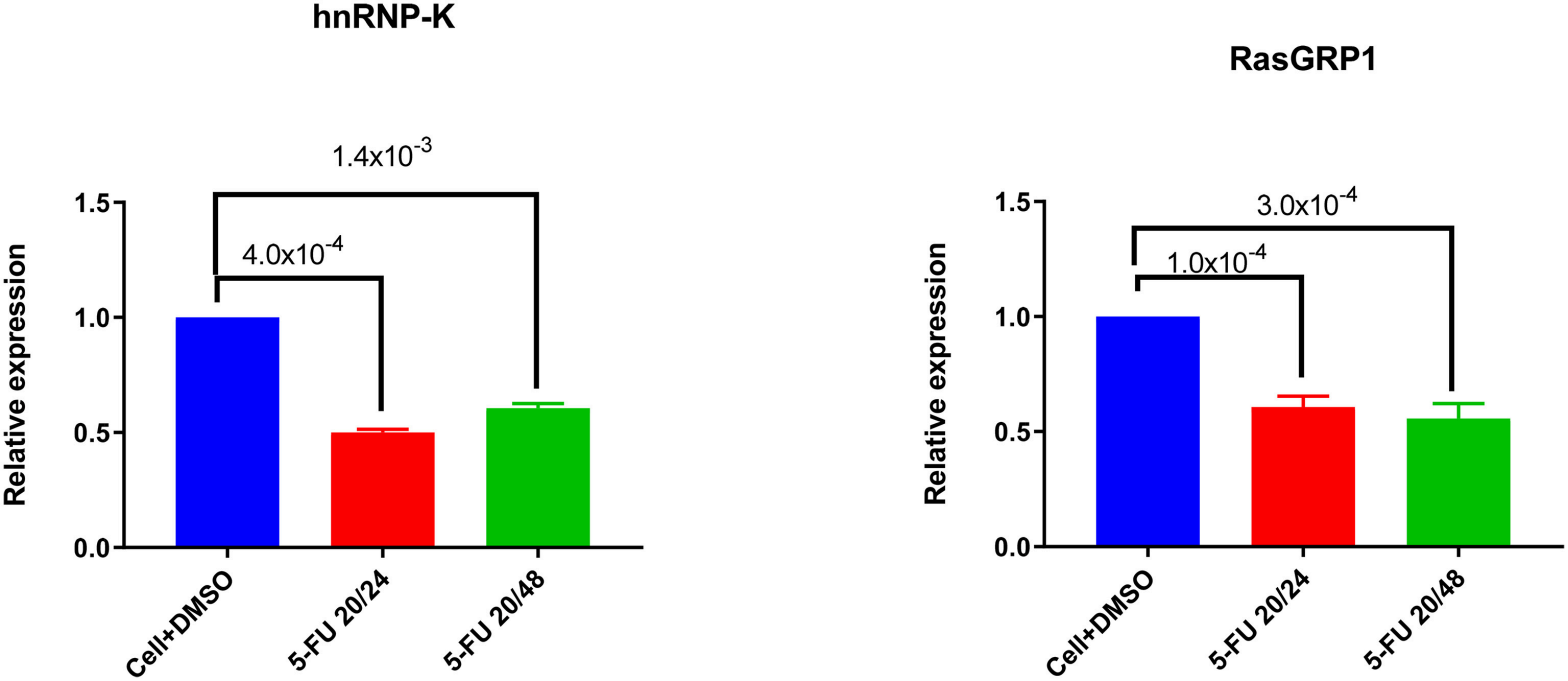
Downregulation of hnRNP-K by 5-FU treatment. 5-FU treatment reduces hnRNP-K expression levels in Jurkat cells. Jurkat cells were treated with DMSO vehicle or 5-FU (20 ng/μl) for 24 or 48 h. *hnRNP-K* **(A)** and *RASGRP1* **(B)** were examined with *GADPH* as loading control.

**Figure 6:**
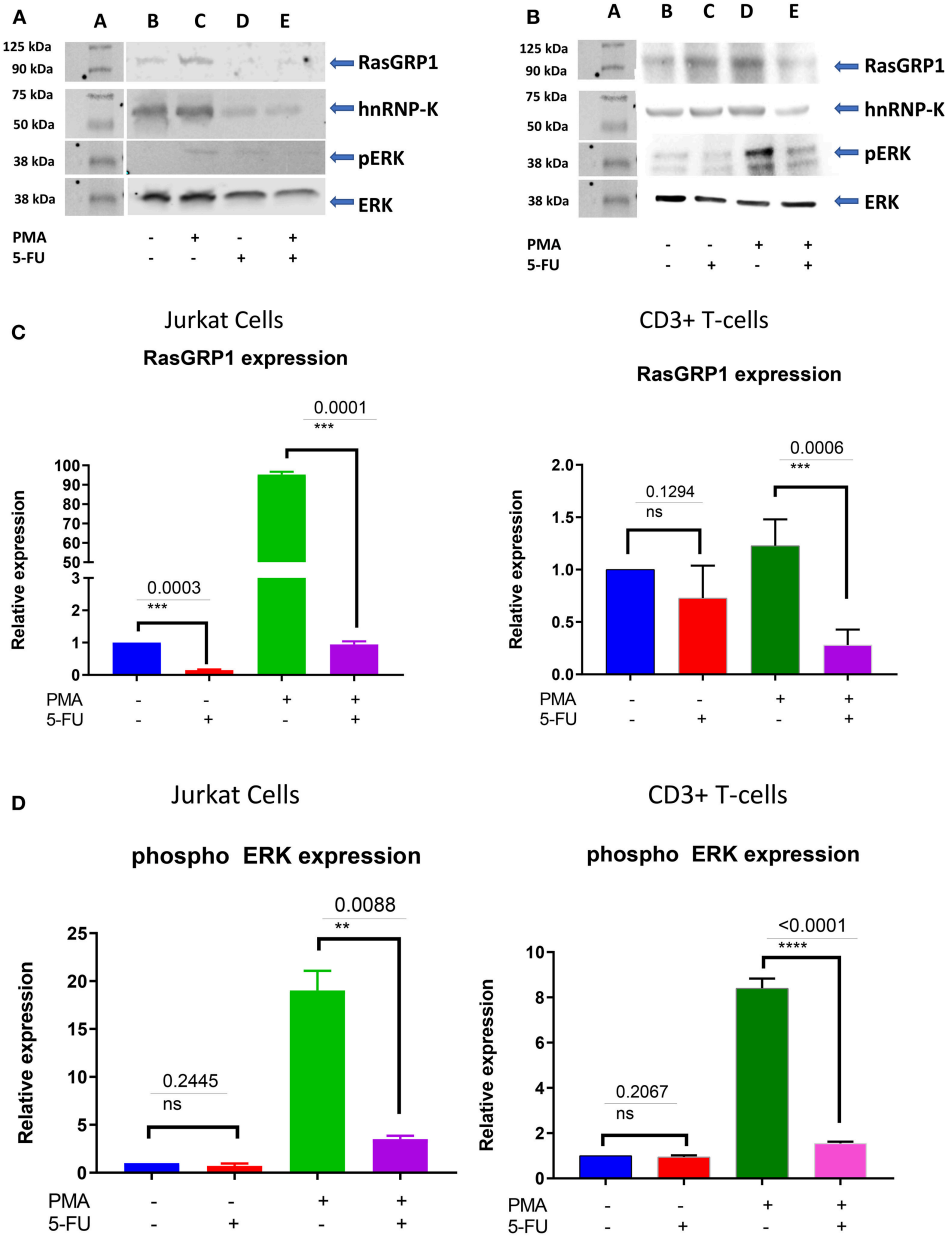
**(A)** RasGRP1 reduction influences the phosphorylation of ERK. 5-FU treatment reduces hnRNP-K and RasGRP1 expression levels in Jurkat and healthy human CD3^+^ T cells. Pretreatment with PMA increases levels of RasGRP1 and phospho-ERK. Inhibition of hnRNP-K with 5-FU decreases levels of RasGRP1 and phospho-ERK, even after PMA stimulation. **(B)** 5-FU treatment reduces hnRNP-K as well as RasGRP1 expression level in primary CD3^+^ T-cells. Pretreatment with PMA induces RasGRP1 expression and leads to phosphorylation of ERK and reduction of RasGRP1; treatment with 5-FU also leads to reduction of phosphorylation of ERK. **(C)** Densitometric analysis for *RASGRP1* normalized to β-actin: primary T-cells and Jurkat cells. Results are presented as relative fold-change following drug treatment with and without stimulation. **(D)** Densitometry analysis for phospho-ERK normalized to β-actin: primary T-cells and Jurkat cells. Results are presented as relative fold-change following drug treatment with and without PMA stimulation. **P < 0.05; ***P < 0.005.

To investigate the role of endogenous hnRNP-K in Jurkat and primary CD3^+^ T-cells, we transiently inhibited hnRNP-K using 5-fluorouracil (5-FU). After 5-FU treatment (48 h), we observed significantly reduced mRNA expression for both hnRNP-K (*P* = 1.4 × 10^−3^; Figure 5A) and *RASGRP1* (*P* = 3.0 × 10^−4^; Figure 5B). 5-FU-induced hnRNP-K downregulation correlated with reduced expression of *RASGRP1* (Figures 6A,B). This result suggests that hnRNP-K plays an important role in *RASGRP1* expression in Jurkat cells as well as in primary T-cells. Furthermore, we observed the reduction of ERK phosphorylation with 5-FU after initial induction with PMA in Jurkat and primary T-cells (Figures 6A–D). It is of note that stimulation with PMA did not influence cell viability (Supplementary Figure 7).

## Discussion

In this study, we fine-mapped our previously reported SLE locus near *RAS guanyl-releasing protein 1* (*RASGRP1*), a lynchpin of T-cell development and the RAS/MAP kinase signaling cascade following antigen exposure. We performed a trans-ethnic meta-analysis of the locus with cohorts of Asian and European descent, followed by multiple lines of bioinformatic analysis of its epigenetic context to prioritize SNPs as candidate causal variants. Experimental testing of the top candidates validated them as plausible variants underlying association of this locus with SLE (and perhaps other autoimmune phenotypes).

We identified two independently associated regions correlated with *RASGRP1* regulation and expression. The first signal lies in *RASGRP1* intron 2, represented by SNPs rs11631591-rs7173565 and rs7170151, which regulate *RASGRP1* expression as eQTLs (esophageal mucosa and skin), enhancers (in CD8^+^ T-cells and thymic and lymphoblastoid cell lines), and as interaction anchors with the nearby *C15orf53* promoter. The SNPs in this region are within a robust enhancer, with the risk alleles (rs7170151-C and rs11631591-C/rs7173565-C) greatly increasing *RASGRP1* expression in multiple tissues (databases) and in Jurkat T-cells (our experiments). Furthermore, this enhancer is targeted by promoter interactions in CD8^+^ and CD4^+^ T-cells, B-cells, and monocytes (62) (Supplementary Figure 3). We also identified another intergenic signal around 60 kb 5′ of *RASGRP1*, at rs9920715, another SNP within promoter-interacting chromatin that acts as an eQTL for *RASGRP1* in B- and T-cell lines (62). However, this SNP did not show enhancer activity in our assays.

Mammalian gene regulatory elements, especially those that are tissue-specific, show high *in vivo* nucleosome occupancy, which can effectively compete with TF binding (63, 64). This nucleosome-mediated restricted access to regulatory information is a key element for inducible or cell type-specific control of gene expression (65). In the current study, we observed strong enhancer activity at rs11631591-rs7173565 or rs7170151 only in Jurkat but not HEK293 cells. Furthermore, our candidate SNPs show allele-specific *RASGRP1* expression, with the risk alleles driving substantially more (~50%) expression than the non-risk alleles. Other studies on numerous complex diseases have demonstrated enrichment of disease-associated loci in cell type-specific regulatory regions of corresponding disease-relevant cell types (58, 66–69). Additional studies now document the direct effects of common variation in enhancer elements on enhancer states (70–73), gene expression (70, 74), and disease (75–79). Risk alleles of rs11631591 also showed significant binding to hnRNP-K protein in an allele-specific manner.

DNA/protein interaction assays demonstrated that hnRNP-K preferentially binds to sequences containing the rs11631591 risk (C) allele. We confirmed this allele-specific binding by EMSA and ChIP DNA sequencing. We only observed allele-specific binding of hnRNP-K at SNP rs116311591-rs7173565, but not at rs7170151 or rs9920715. We also observed that inhibition of hnRNP-K correlates with *RASGRP1* expression and ERK phosphorylation. In fact, expression of *RASGRP1* and hnRNP-K (*P* = 9.8 × 10^−5^; *P* = 1.4 × 10^−2^, respectively) in spleen (Supplementary Figure 8) shows a positive correlation between the risk allele of rs116311591 and both these genes. These data suggest that SNP rs11631591 is a functional SNP and may directly contribute to modulating *RASGRP1* expression. Abnormal expression of *RASGRP1* isoforms will perturb lymphocytes of SLE patients regardless of their clinical disease activity, and may contribute to impaired lymphocyte function and increased apoptosis in SLE patients (19). Abnormal *RASGRP1* expression also induces ERK and JNK phosphorylation in the MAPK pathway, which in turn alters T-cell development, contributes to long-term organ damage, and ultimately increases SLE susceptibility (22, 24, 25). In the present study, we also observed the role of *RASGRP1* expression in the phosphorylation of ERK activity. Altogether, our results indicate increased *RASGRP1* expression correlates with the risk alleles in our functional SLE loci and T-cell dysfunction. However, our study did not examine the differences in *RASGRP1* isoform expression reportedly associated with SLE and correlated with low *RASGRP1* expression (19).

In this study, we characterized the genetic risk of SLE in *RASGRP1*. We also propose a mechanism by which functional SNPs could affect SLE pathogenesis. We identified two functional regions affecting expression and regulation of *RASGRP1* in an intronic region including two SNPs (rs11631591 and rs7170151) and another in an intergenic region harboring SNP rs9920715. All identified SNPs are *RASGRP1* eQTLs and exhibit regulatory potential through enhancer-promoter chromatin interactions. SNP rs11631591 showed T-cell-specific enhancer activity and an allele-specific interaction with hnRNP-K protein. Inhibition of hnRNP-K protein by 5-FU decreased expression of *RASGRP1* in T-cells, suggesting that hnRNP-K plays an important role in *RASGRP1* expression through interactions with the risk genotype of SNP rs11631591. These results are consistent with this SNP being an important factor contributing to SLE pathogenicity.

Heterogeneous nuclear ribonucleoproteins (hnRNPs) represent a large family of nucleic acid-binding proteins implicated in various cellular processes including transcription and translation (24, 80). hnRNP-K is a highly multifunctional protein, with annotated roles in chromatin remodeling, transcription, splicing and translation (80). It is primarily referred to as an RNA-binding protein specific for “poly-C” repeats (81), but it actually prefers single-stranded DNA and can bind to double-stranded DNA (82). hnRNP-K can act as a transcriptional activator or repressor (83); notable examples include transcriptional repression of *CD43* in leukocytes (84) and transcriptional activation of *c-myc* in B-cells (85). Its DNA-binding preference is found to be repeats of the CT motif, separated by several base pairs (82), confirmed by structure determination (86). There are several CT motifs in the immediate environment of rs11631591, whose hnRNP-K binding could be affected by the SNP. It should also be noted that several of the other abundant proteins pulled down by the double-stranded DNA EMSA are primarily annotated as RNA-binding proteins, including hnRNP-M and splicing factor U2AF. Other transcription factors were also abundant, including far upstream element-binding protein 3, supporting the notion that this locus is indeed transcriptionally active.

Taken together, we have identified and mechanistically dissected a lupus risk locus in the 2nd intron of *RASGRP1*, which regulates T- and B-cell development and the MAP kinase pathway. Single SNPs were found to control transcriptional activation and binding to several proteins, including the transcription factor hnRNP-K. Experiments confirmed that both the single base-pair risk-to-non-risk substitutions and pharmacological inhibition of hnRNP-K decreased MAPK signaling in T-cells. Systematic refinement of large GWAS peaks to single SNPs, combined with experimental mechanistic analysis, is critical to understand the genetics of highly multigenic diseases and to drive therapeutic interventions to improve human health.

## Web Resources

Bentham and Morris summary SLE GWAS: http://insidegen.com/.

## Ethics Statement

All sample collections were approved by the Institutional Review Board of the Oklahoma Medical Research Foundation as well as by the collaborating institutions.

## Author Contributions

SN conceived and supervised the project. JM performed the meta-analysis, bioinformatic, and epigenetic analyses, and prepared most of the tables and figures. BS performed most of the experiments and generated experimental figures. CT, YO, and SA ran the association analysis and provided the relevant data for replication of the Japanese samples. JK and BM helped and performed some experiments. CS helped in assembling and analyzing imputed data for association and performed some bioinformatics analysis and also helped in interpreting the results and contributed to the correction of manuscript. CW helped in planning experiments and revising the manuscript. LL provided expertise and helped in interpreting the results. JM, LL, and SN drafted and finalized the manuscript. All authors read and approved the submitted manuscript.

### Conflict of Interest Statement

The authors declare that the research was conducted in the absence of any commercial or financial relationships that could be construed as a potential conflict of interest.
