# Supplementary material for: Mechanistic Characterization of RASGRP1 Variants Identifies an hnRNP-K-Regulated Transcriptional Enhancer Contributing to SLE Susceptibility
Source: Front Immunol. 2019 May 20;10:1066. doi: 10.3389/fimmu.2019.01066 (PMC6536009; doi:10.3389/fimmu.2019.01066)
Supplement: Supplementary file 2 [file Presentation_1.pptx]

## Slide 1
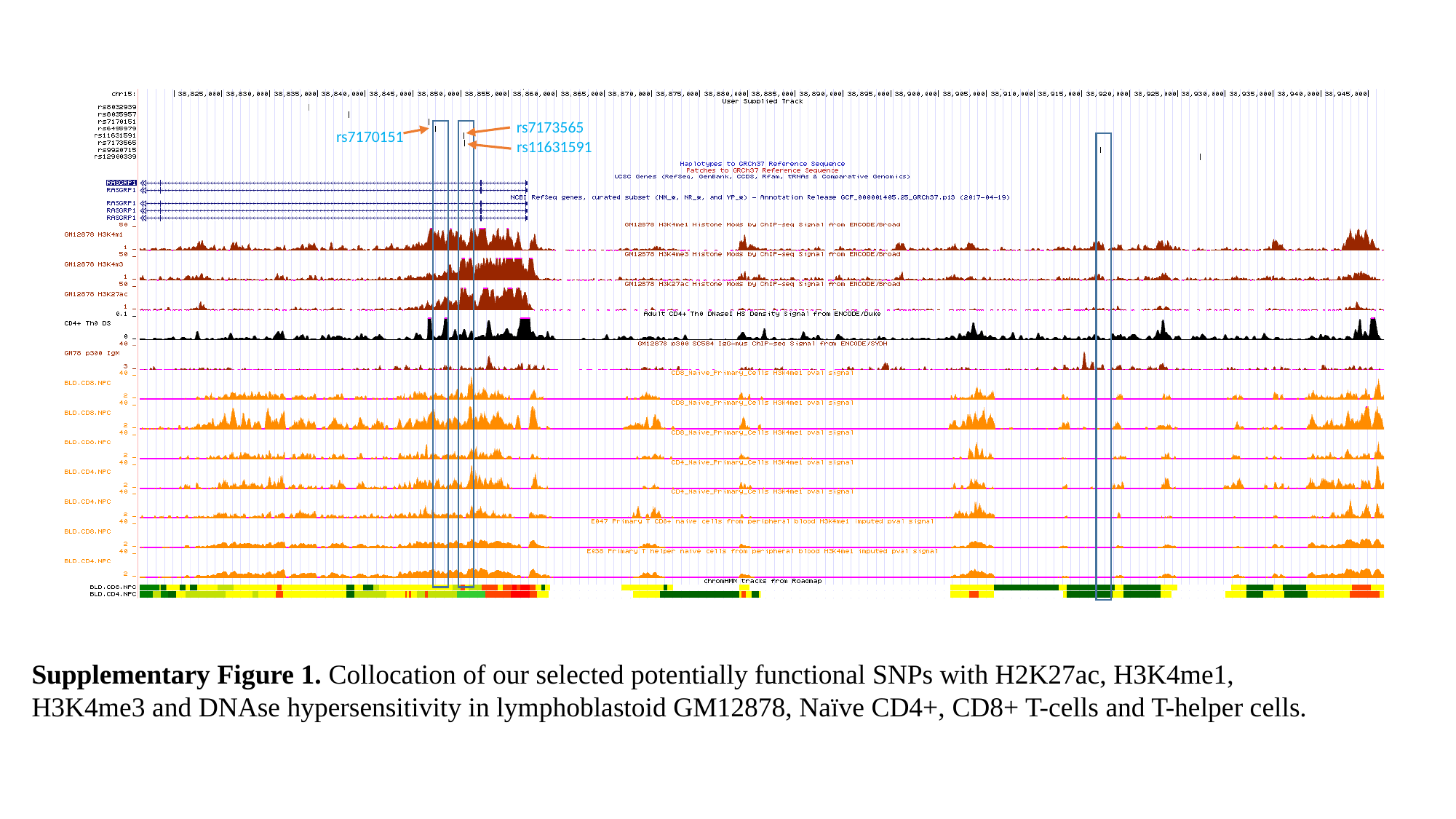

rs7173565
rs7170151
rs11631591
Supplementary Figure 1. Collocation of our selected potentially functional SNPs with H2K27ac, H3K4me1, H3K4me3 and DNAse hypersensitivity in lymphoblastoid GM12878, Naïve CD4+, CD8+ T-cells and T-helper cells.

## Slide 2
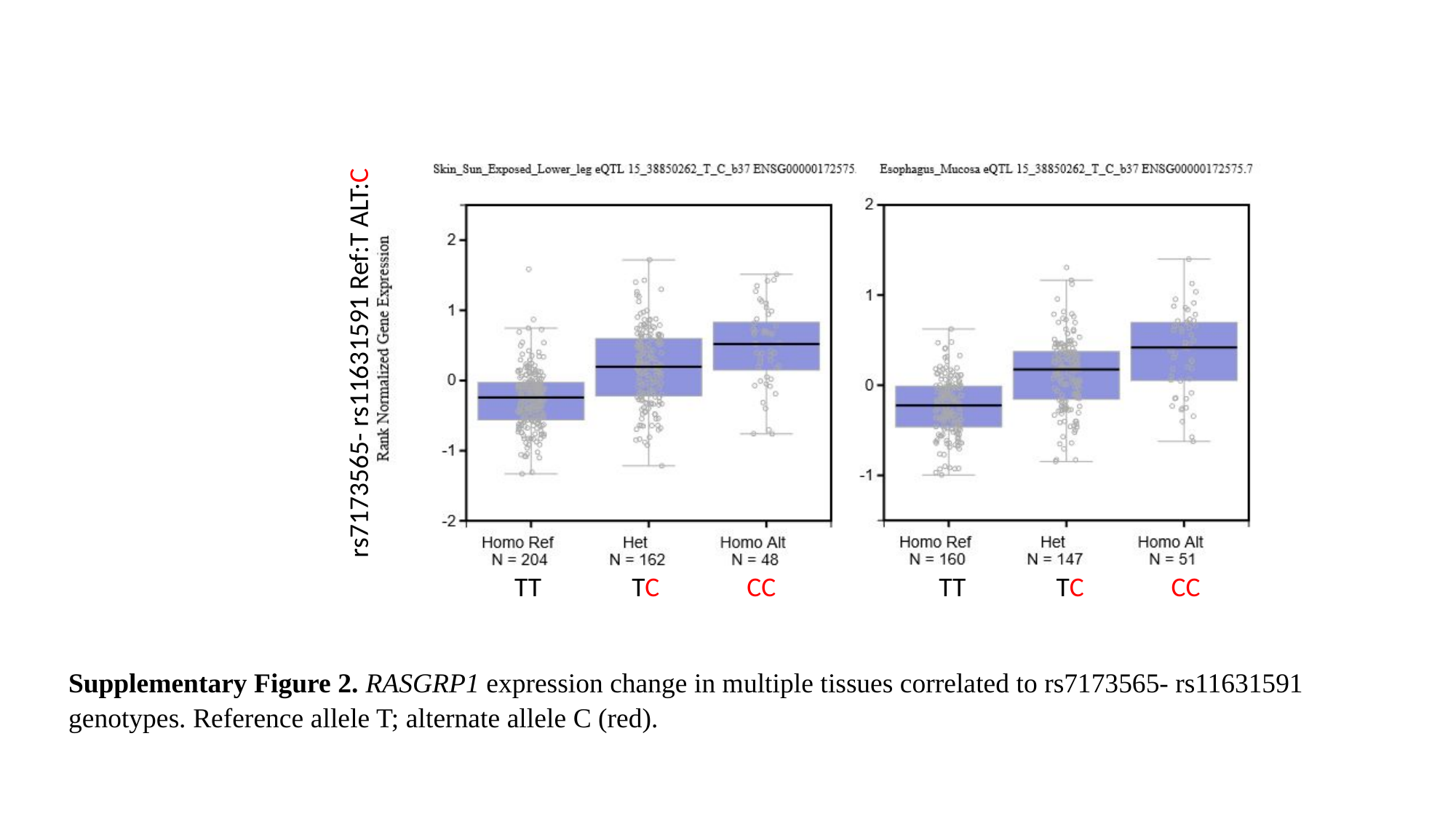

TT
TC
CC
TT
TC
CC
rs7173565- rs11631591 Ref:T ALT:C
Supplementary Figure 2. RASGRP1 expression change in multiple tissues correlated to rs7173565- rs11631591 genotypes. Reference allele T; alternate allele C (red).

## Slide 3
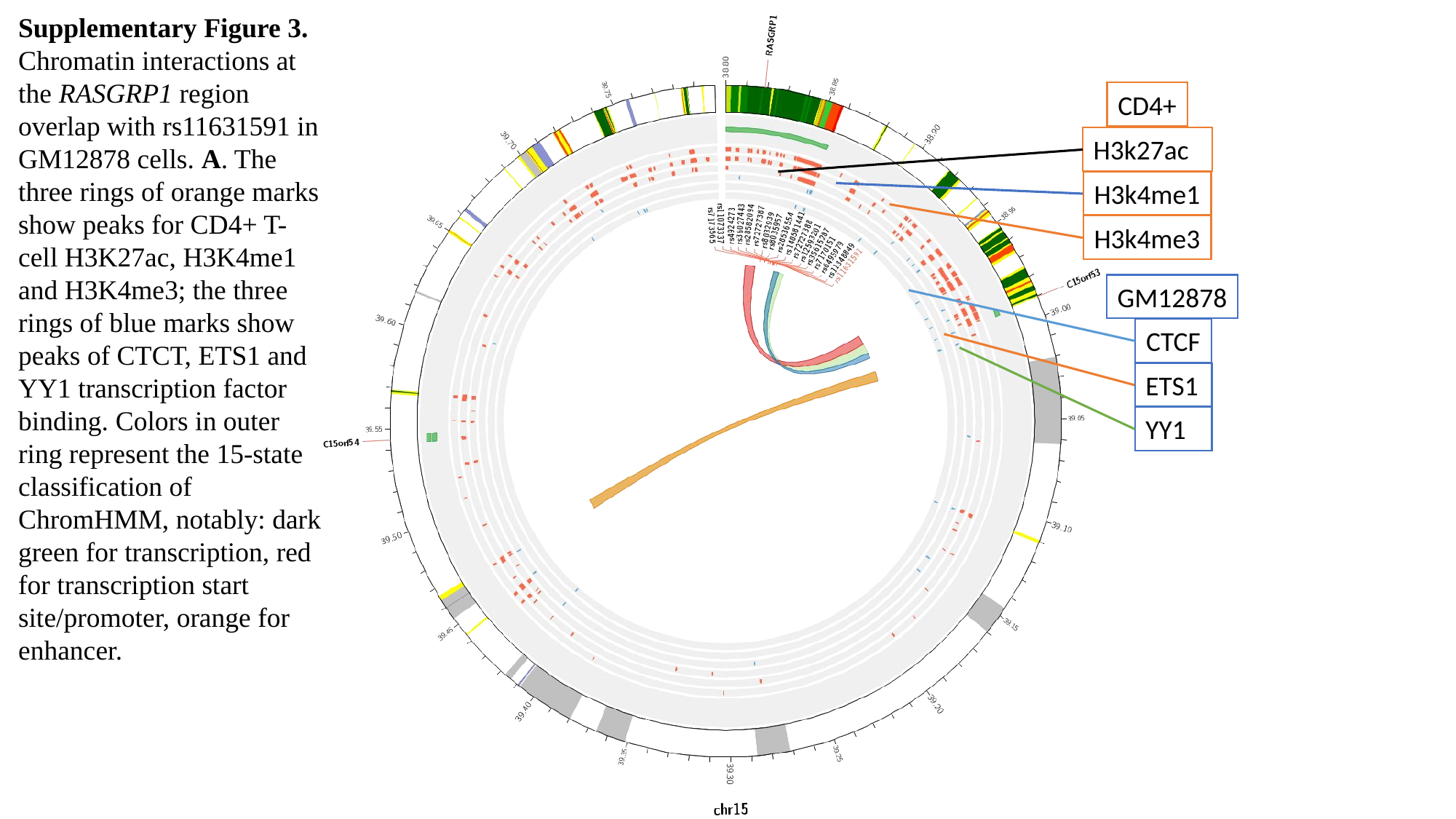

Supplementary Figure 3. Chromatin interactions at the RASGRP1 region overlap with rs11631591 in GM12878 cells. A. The three rings of orange marks show peaks for CD4+ T-cell H3K27ac, H3K4me1 and H3K4me3; the three rings of blue marks show peaks of CTCT, ETS1 and YY1 transcription factor binding. Colors in outer ring represent the 15-state classification of ChromHMM, notably: dark green for transcription, red for transcription start site/promoter, orange for enhancer.
CD4+
H3k27ac
H3k4me1
H3k4me3
GM12878
CTCF
ETS1
YY1

## Slide 4
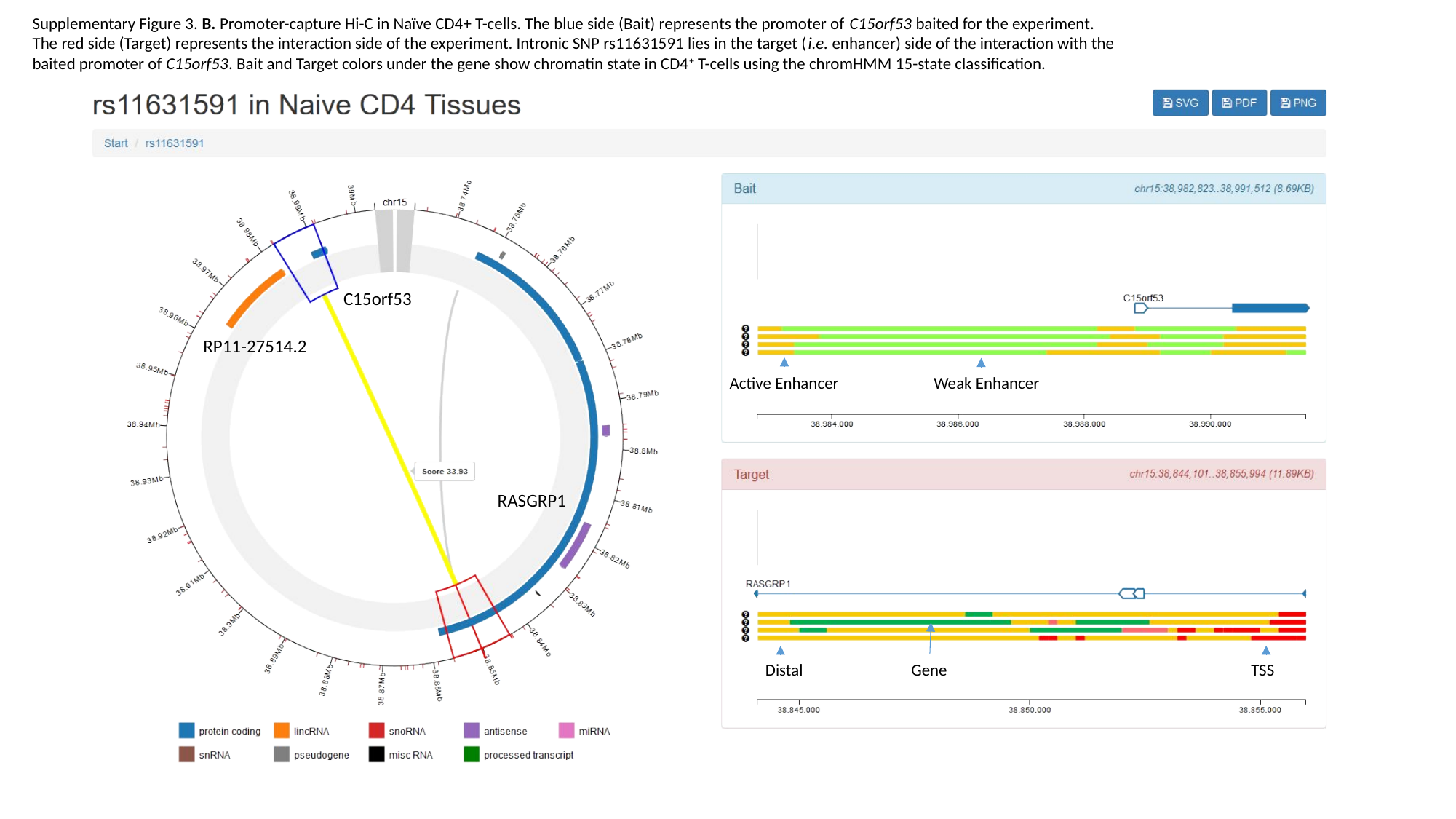

Supplementary Figure 3. B. Promoter-capture Hi-C in Naïve CD4+ T-cells. The blue side (Bait) represents the promoter of C15orf53 baited for the experiment. The red side (Target) represents the interaction side of the experiment. Intronic SNP rs11631591 lies in the target (i.e. enhancer) side of the interaction with the baited promoter of C15orf53. Bait and Target colors under the gene show chromatin state in CD4+ T-cells using the chromHMM 15-state classification.
Active Enhancer
Weak Enhancer
TSS
Distal
Gene
C15orf53
RASGRP1
RP11-27514.2

## Slide 5
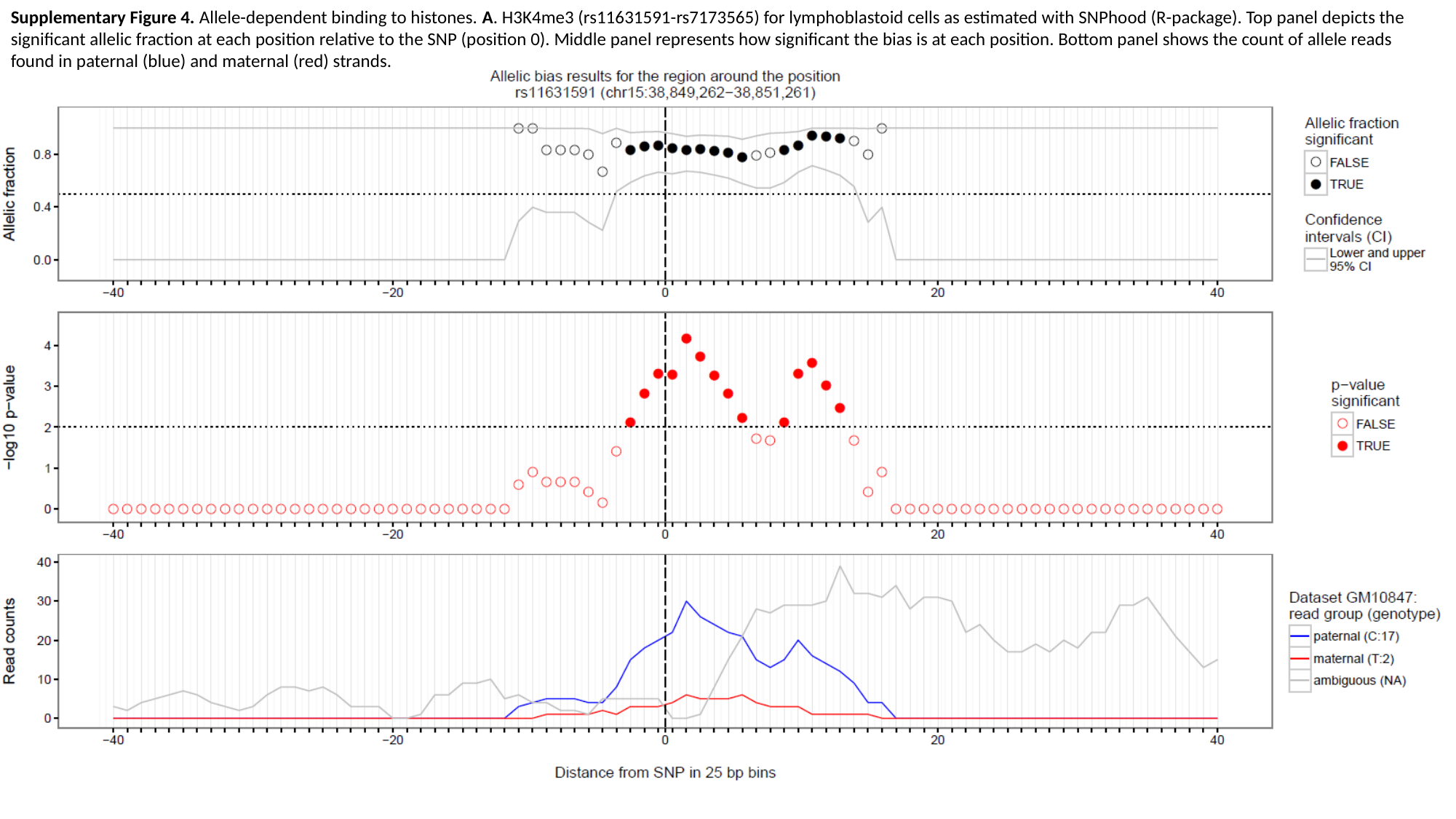

Supplementary Figure 4. Allele-dependent binding to histones. A. H3K4me3 (rs11631591-rs7173565) for lymphoblastoid cells as estimated with SNPhood (R-package). Top panel depicts the significant allelic fraction at each position relative to the SNP (position 0). Middle panel represents how significant the bias is at each position. Bottom panel shows the count of allele reads found in paternal (blue) and maternal (red) strands.

## Slide 6
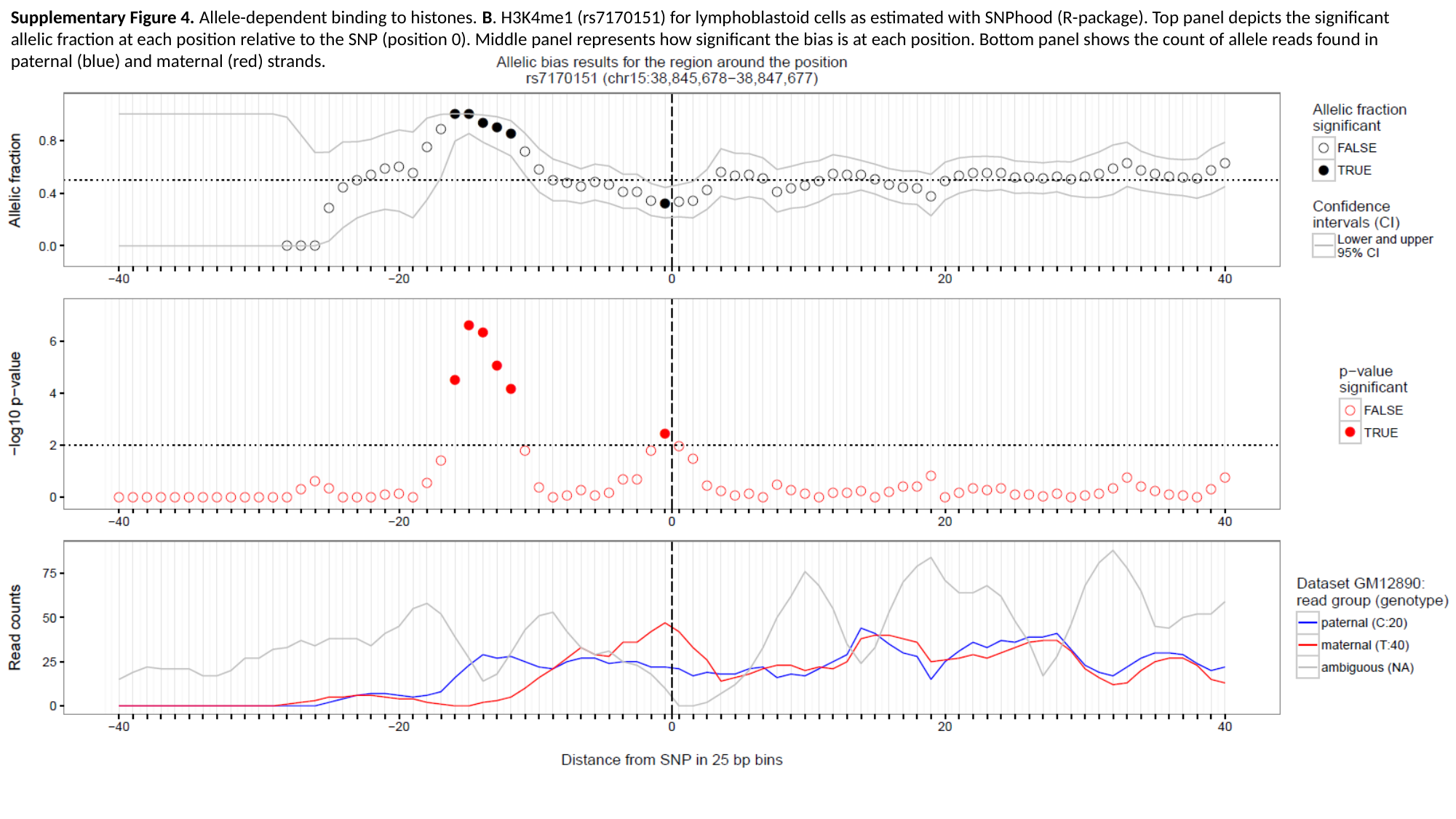

Supplementary Figure 4. Allele-dependent binding to histones. B. H3K4me1 (rs7170151) for lymphoblastoid cells as estimated with SNPhood (R-package). Top panel depicts the significant allelic fraction at each position relative to the SNP (position 0). Middle panel represents how significant the bias is at each position. Bottom panel shows the count of allele reads found in paternal (blue) and maternal (red) strands.

## Slide 7
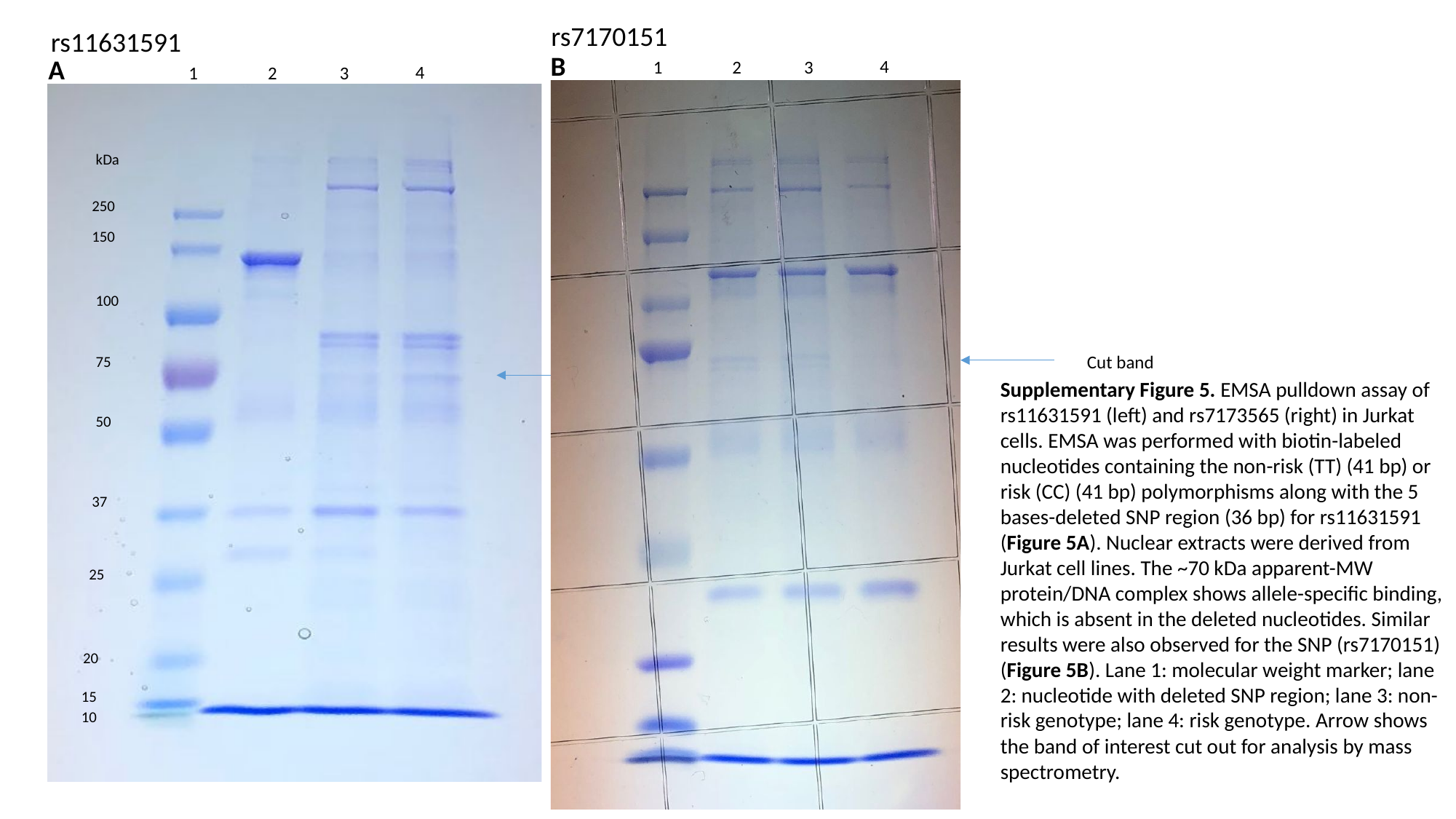

rs7170151
rs11631591
B
A
4
3
2
1
4
3
2
1
kDa
250
150
100
Cut band
75
50
37
25
20
15
10
Supplementary Figure 5. EMSA pulldown assay of rs11631591 (left) and rs7173565 (right) in Jurkat cells. EMSA was performed with biotin-labeled nucleotides containing the non-risk (TT) (41 bp) or risk (CC) (41 bp) polymorphisms along with the 5 bases-deleted SNP region (36 bp) for rs11631591 (Figure 5A). Nuclear extracts were derived from Jurkat cell lines. The ~70 kDa apparent-MW protein/DNA complex shows allele-specific binding, which is absent in the deleted nucleotides. Similar results were also observed for the SNP (rs7170151) (Figure 5B). Lane 1: molecular weight marker; lane 2: nucleotide with deleted SNP region; lane 3: non-risk genotype; lane 4: risk genotype. Arrow shows the band of interest cut out for analysis by mass spectrometry.

## Slide 8
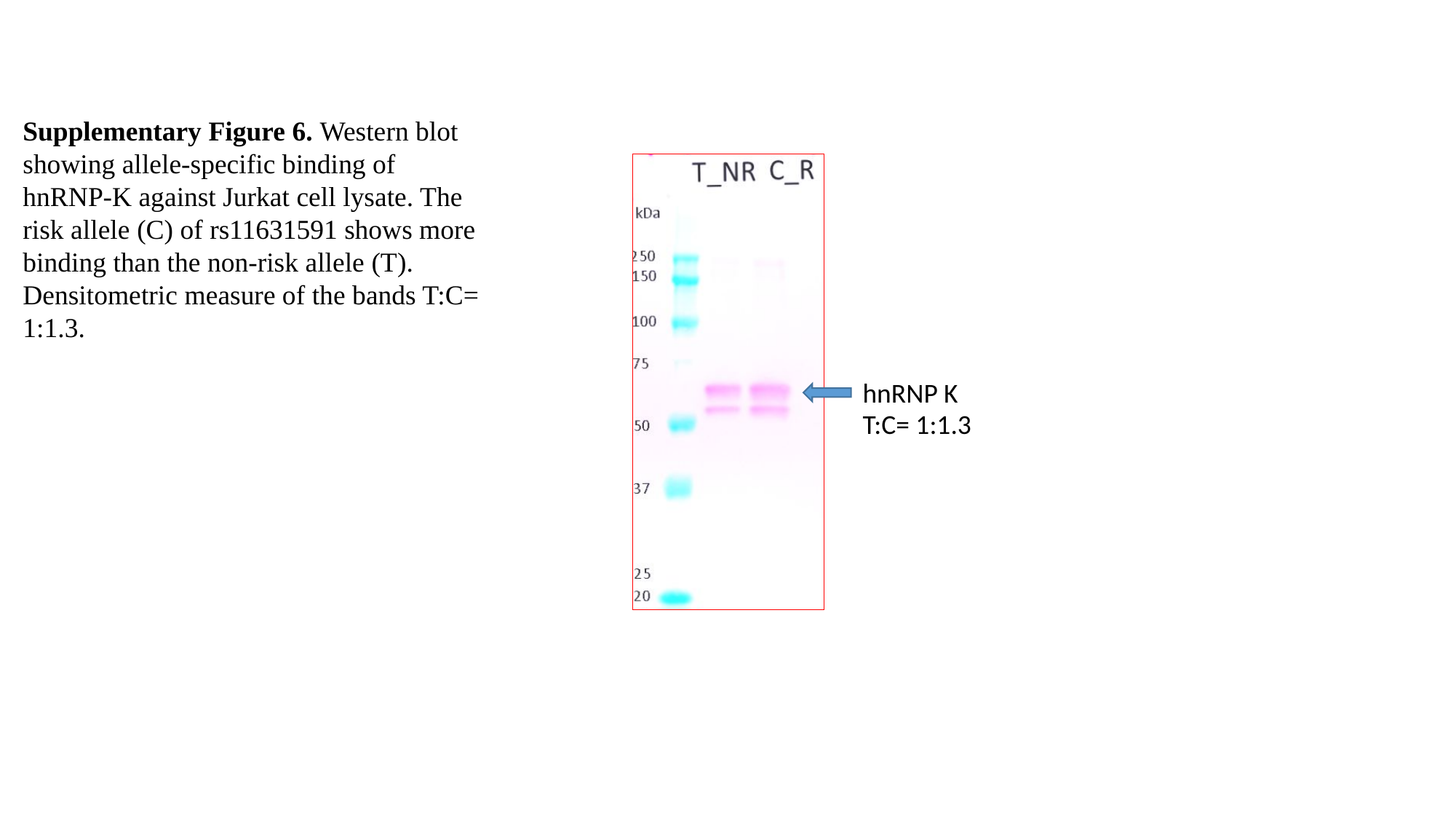

Supplementary Figure 6. Western blot showing allele-specific binding of hnRNP-K against Jurkat cell lysate. The risk allele (C) of rs11631591 shows more binding than the non-risk allele (T). Densitometric measure of the bands T:C= 1:1.3.
hnRNP K
T:C= 1:1.3

## Slide 9
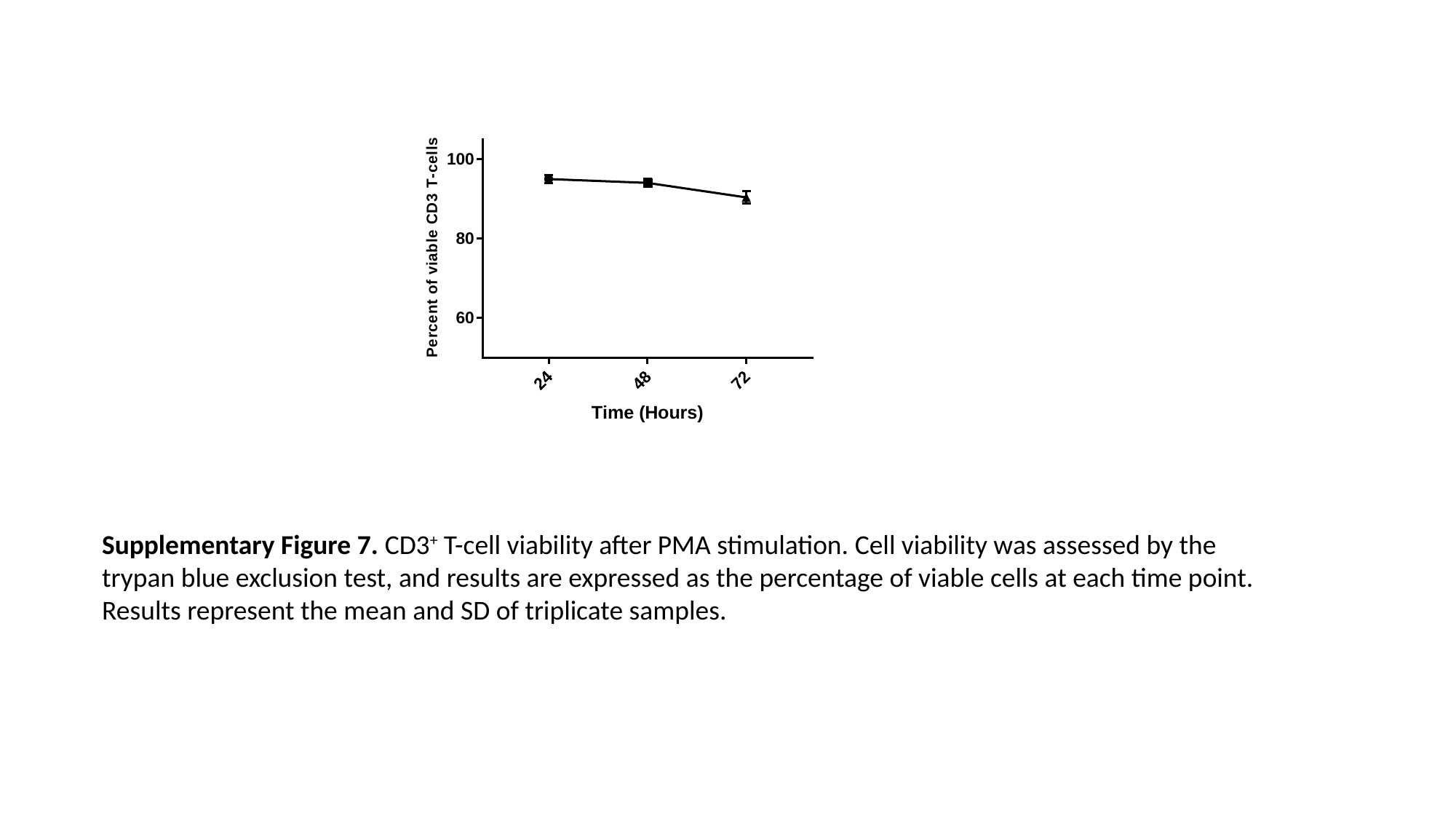

Supplementary Figure 7. CD3+ T-cell viability after PMA stimulation. Cell viability was assessed by the trypan blue exclusion test, and results are expressed as the percentage of viable cells at each time point. Results represent the mean and SD of triplicate samples.

## Slide 10
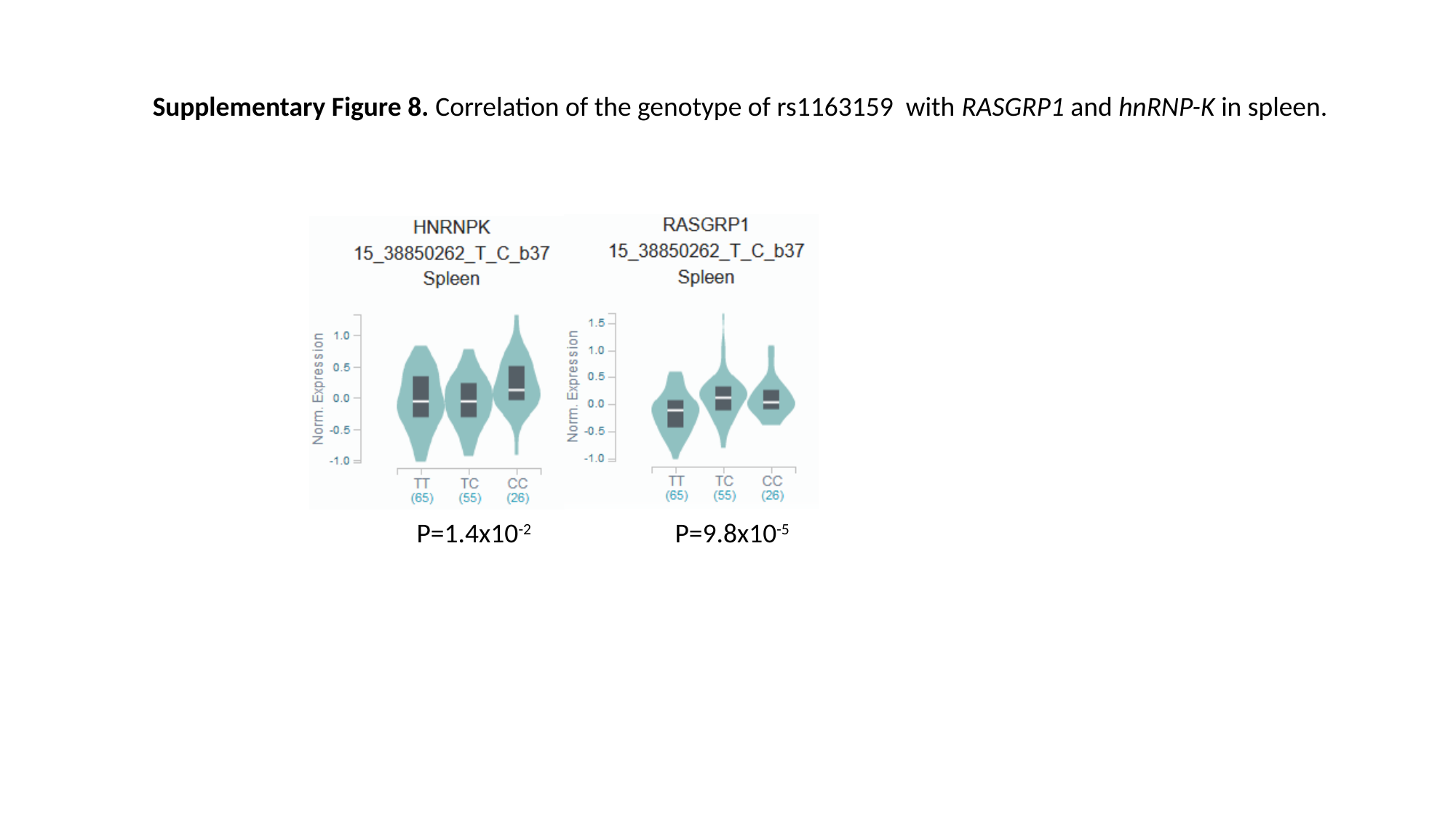

Supplementary Figure 8. Correlation of the genotype of rs1163159 with RASGRP1 and hnRNP-K in spleen.
P=1.4x10^-2^
P=9.8x10^-5^

## Slide 11
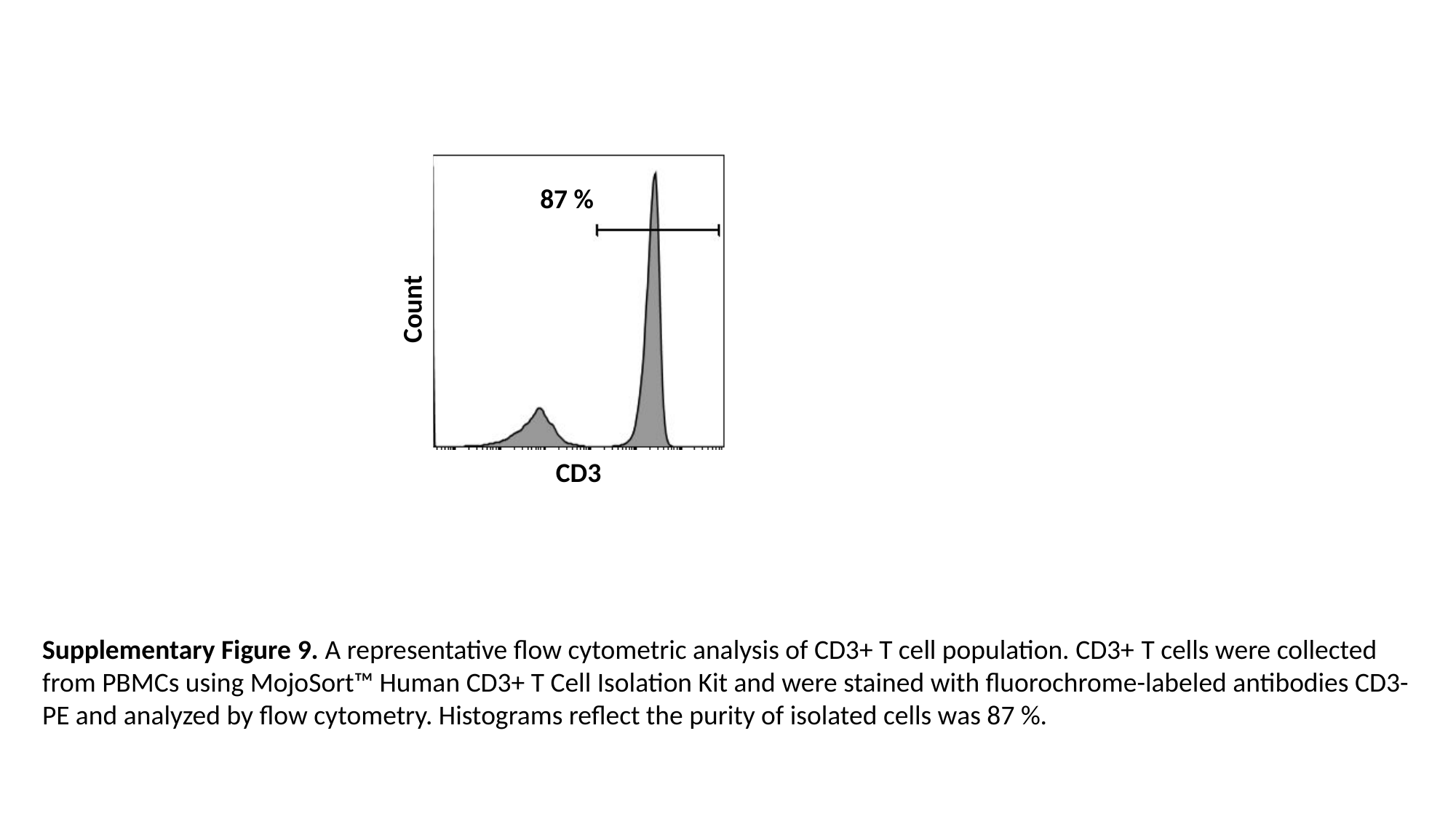

87 %
Count
CD3
Supplementary Figure 9. A representative flow cytometric analysis of CD3+ T cell population. CD3+ T cells were collected from PBMCs using MojoSort™ Human CD3+ T Cell Isolation Kit and were stained with fluorochrome-labeled antibodies CD3-PE and analyzed by flow cytometry. Histograms reflect the purity of isolated cells was 87 %.
